# Unified Roadmap for ZIF-8 Nucleation and Growth: Machine Learning Analysis of Synthetic Variables and Their Impact on Particle Size and Morphology

**DOI:** 10.1021/acs.chemmater.4c01069

**Published:** 2024-05-20

**Authors:** Juan A. Allegretto, Diego Onna, Sara A. Bilmes, Omar Azzaroni, Matías Rafti

**Affiliations:** † Laboratory for Life Sciences and Technology (LiST) Faculty of Medicine and Dentistry, Danube Private University, 3500 Krems, Austria; ‡ Instituto de Investigaciones Fisicoquímicas Teóricas y Aplicadas (INIFTA), Departamento de Química, Facultad de Ciencias Exactas, Universidad Nacional de La Plata, CONICET, CC 16 Suc. 4, La Plata B1904DPI, Argentina; § Instituto de Química Física de los Materiales Medio Ambiente y Energía (INQUIMAE), CONICET-Universidad de Buenos Aires, Buenos Aires C1053ABH, Argentina; ∥ Departamento de Química Inorgánica Analítica y Química Física, Facultad de Ciencias Exactas y Naturales, Universidad de Buenos Aires, Buenos Aires C1053ABH, Argentina

## Abstract

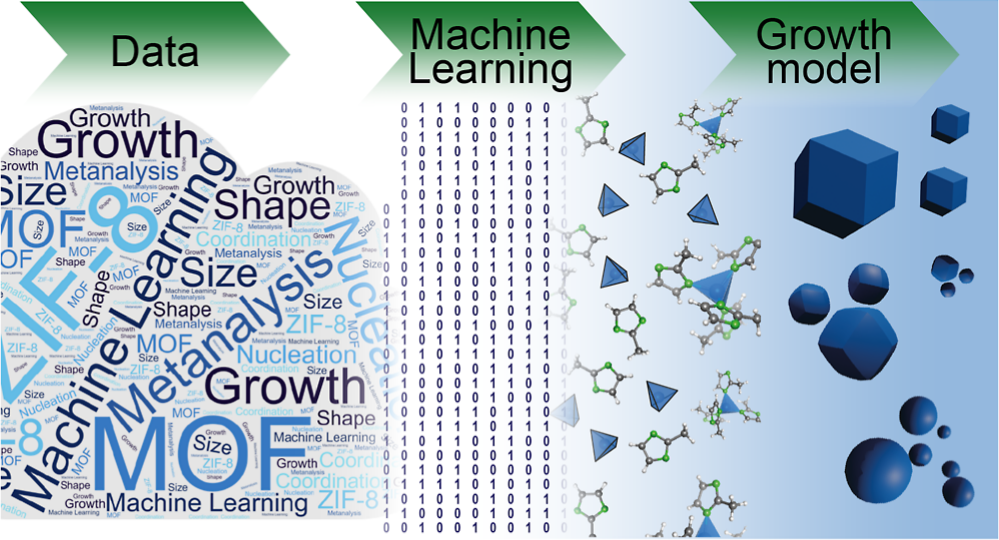

Metal–organic frameworks (MOFs) have settled in the scientific community over the last decades as versatile materials with several applications. Among those, zeolitic imidazolate framework 8 (ZIF-8) is a well-known MOF that has been applied in various and diverse fields, from drug-delivery platforms to microelectronics. However, the complex role played by the reaction parameters in controlling the size and morphology of ZIF-8 particles is still not fully understood. Even further, many individual reports propose different nucleation and growth mechanisms for ZIF-8, thus creating a fragmented view for the behavior of the system. To provide a unified view, we have generated a comprehensive data set of synthetic conditions and their final outputs and applied machine learning techniques to analyze the data. Our approach has enabled us to identify the nucleation and growth mechanisms operating for ZIF-8 in a given sub-space of synthetic variables space (chemical space) and to reveal their impact on important features such as final particle size and morphology. By doing so, we draw connections and establish a hierarchy for the role of each synthetic variable and provide with rule of thumb for attaining control on the final particle size. Our results provide a unified roadmap for the nucleation and growth mechanisms of ZIF-8 in agreement with mainstream reported trends, which can guide the rational design of ZIF-8 particles which ultimately determine their suitability for any given targeted application. Altogether, our work represents a step forward in seeking control of the properties of MOFs through a deeper understanding of the rationale behind the synthesis procedures employed for their synthesis.

## Introduction

Metal–organic frameworks (MOFs) were introduced in the early 90s and the number of research projects and reported structures have dramatically increased ever since.^1−3^ Due to their enormous versatility in terms of constituents and possible structures, MOFs are rich in diversity,^4,5^ which makes the task of selecting suitable candidates for a given use from the +90.000 already reported (and over half million predicted), by no means attainable with experimental work within reasonable time frames.^6^ To overcome this issue, nowadays common in materials science, big data and machine learning (ML) approaches can be employed to establish properties and applications of different materials, including MOFs.^7−13^

A successful ML approach relies on the existence of appropriate databases.^12,14−16^ It can provide interesting alternatives for the identification of suitable candidates for various applications^17^ or the determination of key structural and functional features, and it has been successfully employed in MOF research over the last years.^18−25^ Another possible alternative is to use ML for systematization and validation of multiple reported synthesis methods for a particular material, as it was recently demonstrated for an archetypal Copper-trimesic acid MOF, HKUST-1.^26−28^ Another example is the use of linear models to investigate the formation of defects on UiO-66 MOF and their impact on its catalytic and adsorptive performance.^29^ This approach provided valuable insights into optimizing defect formation through various synthetic parameters. It becomes clear then that ML and big data are powerful tools to obtain new insight and unveil underlying relations among the synthesis parameters used for synthesizing MOFs and for determining their suitability for a given application.

The zeolitic imidazolate frameworks (ZIFs) subclass of MOFs composed of imidazole-based linkers tetrahedrally coordinating metallic ions, such as Zn^+2^ or Co^+2^, stand out due to their suitability for diverse applications and straightforward synthesis methods.^30^ Among the most studied members of the ZIF family is ZIF-8, based on Zn^2+^ ions and 2-methyl imidazolate anions (mIm^–^). ZIF-8 features a crystalline structure with permanent and hydrophobic microporosity (11.6 Å pore diameter), with BET surface areas that can go up to 1950 m^2^ g^–1^ (maximum value estimated from crystallographic data); good chemical and thermal stability, retaining its porous structure at temperatures below 450 °C; and good mechanical stability (Young’s modulus ∼3–4 GPa).^30−33^ These interesting properties triggered intense activity during the past decades, resulting in multiple applications using ZIF-8, such as gas- and liquid-phase separations,^34,35^ vaccine-related technologies,^36^ energy conversion,^37,38^ plasmonics,^39,40^ and drug delivery.^41,42^

The above-discussed relevance of MOFs, and ZIF-8 in particular, across different fields of application resulted in a diversification of synthesis methods and led to somehow contradictory hypotheses related to the formation mechanisms. There are individual reports exploring intensively certain subregions of the synthesis conditions space and proposing operating mechanisms, mostly based on classic nucleation and growth theories (CNT).^43,44^ In the case of ZIF-8, some reports argue that, for example, crystalline nuclei are formed in the initial stages;^45^ it has also been proposed that nucleation takes place by internal reorganization of metastable phases which arise upon precursor mixing.^46^ Different growth mechanisms were also proposed; some reports show that growth proceeds via the addition of individual ions and linkers^47^ or by the addition of small units.^48^ Aggregative growth was also reported as one of the mechanisms ruling ZIF-8 formation.^49^ This variety reflects in a dispersion on the reported results across the literature regarding structural and functional properties, such as the final morphology (from perfectly faceted to quasi-spherical particles) and particle size (from a few tens to hundreds of nm). It becomes clear the need for a methodology to systematize and summarize results of reported procedures and to extract insights or patterns from previous data.

We hereby aim to gain a deeper insight into nucleation and growth mechanisms operating during ZIF-8 formation by resorting to the numerous published reports, which upon curation following objective criteria, were compiled into an ad-hoc data set for the application of ML tools.^50^ In doing so, time-consuming high-throughput screening of experimental synthetic conditions could be avoided, and also important, relevant, and reliable results can be highlighted for its use in applications demanding materials with particular features. Based on the conducted analysis, we provide a unified roadmap for understanding different mechanisms operating during ZIF-8 formation under the most frequent synthetic conditions. Additionally, using meta-analysis we identify the role and relative relevance of different synthetic conditions employed across the literature on the size and morphology of ZIF-8 particles obtained.

## Methodology on Data Set Construction, Analysis, and Data Extraction

The data set for this work was generated by systematic extraction of information regarding synthetic conditions and properties of ZIF-8 particles from the literature. The full description of the criteria adopted for such selection can be found in the Supporting Information file. The entire data set is openly available in Zenodo.^50^ First, relevant sources of information that provide the synthesis conditions and properties of ZIF-8 particles were identified. It is important to ensure the data quality, as the findings depend on these sources. Then, the information was thoroughly and systematically extracted and organized. The data set was analyzed to verify the accuracy and reliability of the extracted information, i.e., thus ruling out common errors arising from mistyping or scale mismatch from microscopy images. Certain relevant parameters were extracted from these articles, describing the synthesis conditions employed and properties of the resulting materials. These include the source and amount of zinc, 2-methylimidazole and solvent, temperature, reaction time, stirring conditions, and information about the resulting material characteristics such as particle size, morphology, and porosity. The morphology of the particles were grouped into three categories based on TEM/SEM images, namely, Faceted, Poor-faceted, and quasi-spherical particles.

**Figure 1:**
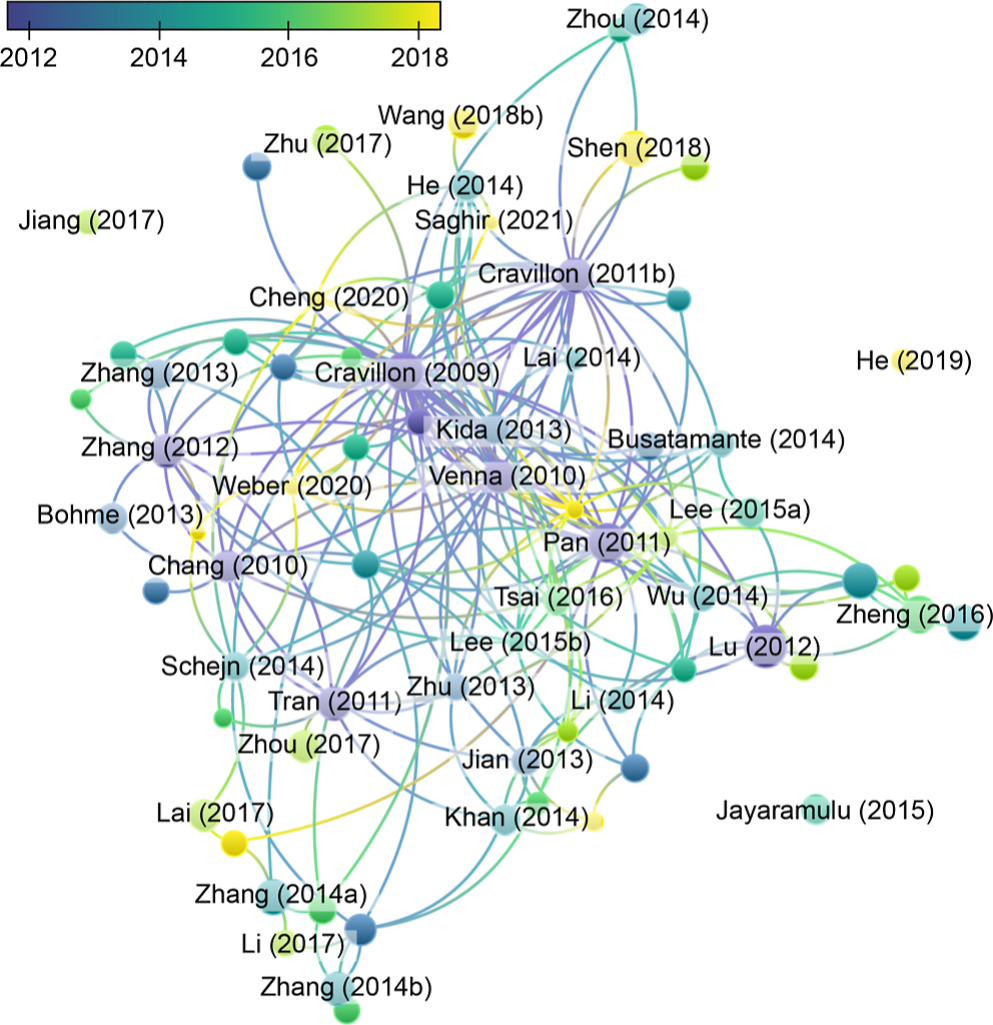
Interconnectivity of documents employed for the data set is shown by lines between nodes. Node diameter is proportional to the citation number, and the date of publication is indicated using a color scale. Plot generated by VOSviewer.^51^

As schematized in Figure S1 in the Supporting Information, the data set for this work was generated by collecting research articles from Scopus, using different combinations of search keywords: ZIF-8, Zeolitic, Framework, and MOF, giving a total of 3440 entries. After this, duplicates and review articles were identified and removed for a final number of 2248 entries. A total of 165 articles were hand-picked from those entries, by title + abstract analysis, starting with the highest citation score and covering the entire set of entries. The final data set thus generated includes representative research covering 15 years between 2006 and 2021. As can be seen from Figure 1, many of the sources used to extract synthetic conditions are interconnected, as new research projects often build on seminal works. While this approach can be helpful, it also means that only a limited portion of the synthetic conditions space (also known as the chemical space) is typically explored. Even further, the lack of reports including negative results (i.e., a synthesis that did not lead to the formation of ZIF-8) also influences the explored conditions and establishes unintentional biases.^52,53^ These factors must be taken into account when extracting information from literature; by no means the absence of data points in a given set of conditions or portion of the chemical space can be thought of as an indicator of the nonviability of those conditions toward ZIF-8 synthesis. From the above discussion, it can only be further stressed the need for a skeptical vision from experts to interpret the results from these sparse and small subsets. It is important to note that the automatization of data extraction from the literature is by no means trivial due to its unstructured organization.

Controlling nucleation and growth processes and understanding the mechanisms involved, is one of the most challenging tasks for advanced materials synthesis, and it has been tackled from multiple perspectives^43^ and for different cases, such as sol–gel-based materials,^54^ zeolites,^55^ and later extended to MOF materials.^44^ For classical nucleation theory (CNT), the initial process toward the formation of a crystal is nucleation (the reader is referred to the numerous reports specifically discussing nucleation and growth theories^43,44,54−56^ and the summary provided in the Supporting Information). Nucleation is highly influenced by synthetic conditions and is typically addressed in terms of the supersaturation of the system.^56^ Supersaturation is ultimately related to the chemical work Δμ resulting from molecular transport-based phenomena taking place, which can be written as Δμ = *k*_B_*T* ln(*C*/*C*_0_), where *k*_B_ is the Boltzmann constant, *T* is the absolute temperature, and *C* and *C*_0_ are the concentration of supersaturated and saturated solutions, respectively. Due to such a strong influence of temperature and concentration on the above-discussed processes, and to be able to establish a straightforward comparison between experimental data, solvothermal synthesis (i.e., experiments carried in hermetic vessels, at temperatures higher than the solvent boiling temperature) will not be included in the present analysis.^57^ In addition, the data was filtered under the condition of reaction times longer than 10 min to guarantee homogenization in the mixing and completion of the first nucleation stages, such as Crystalline Primary Particle formation (vide infra). Synthesis procedures including chemical modulators (i.e., species affecting the availability or coordination environment of at least one of the MOF constituents and influencing the nucleation and growth process) have also been widely used to alter the ZIF-8 synthesis; since they affect the underlying mechanisms of ZIF-8 formation in fundamentally different ways (and therefore not easy to capture through a ML approach), reports employing chemical modulators were not included in our analysis.^48,58−60^

## Results and Discussion

### Nucleation and Growth Mechanisms of ZIF-8

**Figure 2:**
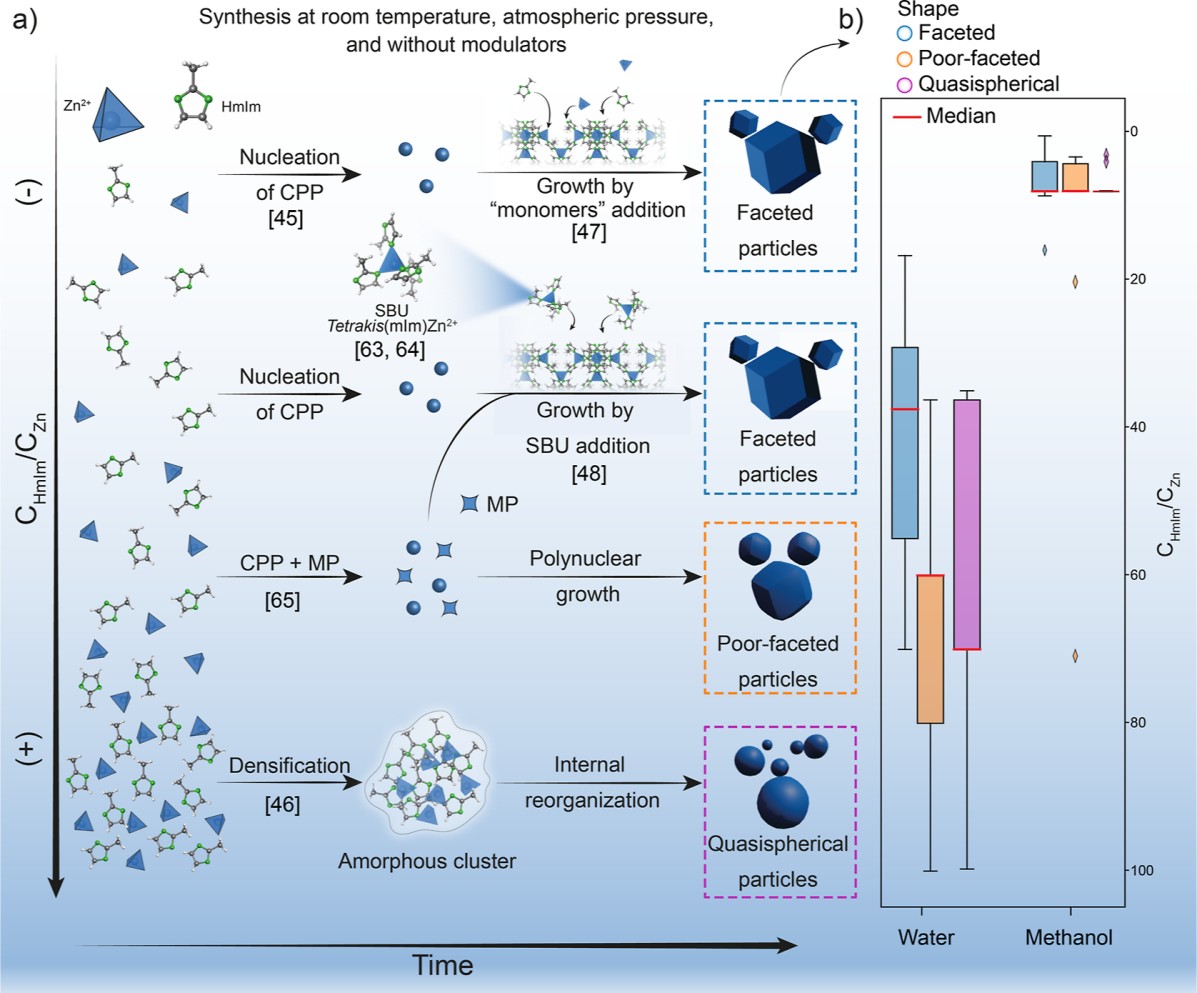
(a) Schematic representation of the underlying mechanisms behind nucleation and growth (left to right) of ZIF-8 from low to high (top to bottom) *C*_HmIm_/*C*_Zn_ molar ratios and final morphology of the particles. (b) Reported morphology of particles obtained at each *C*_HmIm_/*C*_Zn_ molar ratio using water (left) and methanol (right) as the solvent. CPP: Crystalline Primary Particle; SBU: Secondary Building Unit; MP: Metastable Phase. Numbers in Square brackets are the references for the individual reports.

Nucleation and growth mechanisms of ZIF-8 have been studied during the past decade by different approaches; however, most of the studies employed different synthetic conditions for the synthesis, meaning different supersaturation regimes, among other variables such as temperature, counterions, solvent, time, etc. Individually, each study provides information about the underlying mechanism operating in the proximity of the synthetic conditions explored. A more comprehensive understanding of nucleation and growth of ZIF-8 can be reached by organizing the reports according to the supersaturation conditions employed, as depicted in Figure 2a. Since the supersaturation condition is not easy to establish from experimental synthesis conditions reported, the discussion will be carried out in terms of the molar ratio upon a mixture of MOF constituents solutions (zinc, hereinafter Zn, for simplicity, and 2-methylimidazole HmIm) and the solvent (Sv) (Zn/HmIm/Sv). It is important to highlight that concentration values employed correspond to nominal concentrations, that is, not taking into account any pH speciation or similar.

At low concentrations and low excess Zn/HmIm molar ratio (excess molar ratio refers to any ratio higher than stoichiometric 1:2 ratio), CNT can be considered to hold; the material nucleates via the formation of crystal nuclei. In line with the above-discussed ideas, small, crystalline, and well-faceted ZIF-8 particles (with a radius of gyration *R*_g_ ∼20 nm, equivalent to a 50 nm diameter sphere) were reported during the first ∼300 s of synthesis, which were called crystalline primary particles (CPPs) by Cravillon et al. (see the upper left box in Figure 2a).^45^ The authors have also identified that beyond 300 s the coalescence process starts (although individual particles retain their size and shape), indicating that particle growth took place during the first minutes of synthesis. From the thermodynamic point of view, classical growth mechanisms operate and yield morphologies that minimize the total Gibbs surface free energy. At the same time, a kinetic analysis would suggest that, for the growth process to take place, MOF precursors must diffuse and reach the surface of the growing crystal and form coordination bonds. Both steps (diffusion and coordination) could be the limiting step, depending on synthetic conditions. For low supersaturation regimes, i.e., low linker excess and low concentration of reactants, individual units (ZIF-8 precursors) coordinate onto energetically favorable points, in the so-called mononuclear regime (see the upper left panel in Figure 2a).^47^ It was proposed that the process involves partial coordination of imidazolate linkers with both CPP and solvent, during the formation of the ordered ZIF-8 structure, which ultimately stabilizes the developing pore structure.^47,61−63^ This process results in the formation of particles with well-defined edges, favoring the densest (110) crystalline planes and thus leading to the classic rhombic dodecahedron shape (see Figure S2 for the classification of the morphology of ZIF-8 particles). By increasing the concentrations or the HmIm excess, CPPs were found to grow by the addition of small clusters of 1.1 nm rather than by coordination of individual precursors (see the second row in Figure 2a).^48^ In one of the reports in which such a mechanism is described, the authors suggested that growth took place through monomer addition mechanism; however, based on the classification herein employed, it corresponds rather to a polynuclear regime. The proposed clusters were later proved to correspond to the unit *tetrakis*(mIm)Zn^2+^,^64^ which are usually referred in the literature as SBUs (secondary building units). In a later report, it was found that although the *tetrakis*(mIm)Zn^2+^ cluster is the most stable, other Zn^2+^ clusters are present at early nucleation stages and are likely to be involved in the overall process.^65^

Classical models described above assume that during growth no change in the crystalline phase of the nuclei occurs; however, under a given condition these assumptions might not hold. One of the models that account for nonclassical growth is the Ostwald step rule, in which crystallization of the same final unit can take place by successive formation of intermediate phases. In this model, the phase with the lower formation energy is the first to appear, instead of the most thermodynamically favored, and then, due to transformations such as dissolution–recrystallization or internal reorganization processes, the final product is obtained. This kind of mechanism was also observed for ZIF-8 formation; amorphous phases together with ZIF-8 CPPs were found when either concentration or HmIm excess increases,^65,66^ leading to poor-faceted yet crystalline particles (see the middle region in Figure 2a) where growth took place at the expense of the metastable phases during the first 60 min. The dynamics observed are highly dependent on the solvent employed, as we will discuss later on. For example, for aqueous synthesis, the metastable phases were identified as Zn(OH)_2_, Zn(OH)(NO_3_)(H_2_O), and Zn_5_(OH)_8_(NO_3_)_2_(H_2_O)_2_.^67^ When using high molar excess conditions, a three-step nucleation mechanism for ZIF-8 which proceeds via the formation of metastable phases was reported.^46^ Here, an initial phase separation into poor- and rich-solute regions was revealed, followed by the formation of an amorphous cluster by densification, as shown in the bottom row of Figure 2a. Next, the formation of particles through internal reorganization of the amorphous cluster occurs. The authors also detected sporadic formation of small clusters (<5 nm) before phase separation. In addition, they reported that not all rich-solute regions turn into crystalline phases; some of them were observed to redissolve. There is a strong resemblance between the above-discussed mechanisms and those typically seen for zeolites, as the Nanoslab model for example, which reinforces the similarity between these two families of porous materials.^55^

Based on the points above discussed, it can be safely assumed that ZIF-8 formation does not take place by a single mechanism; there are several pathways by which the material can be formed, depending on the synthesis conditions employed. Furthermore, the final characteristics of the particles (i.e., size and morphology) also rely on the operating mechanism, especially for crystalline materials. For example, particles that have growth by monomer addition, as schematized in the first row of Figure 2, will typically present facets, as the growth proceeds in an organized manner; on the other hand, as the mechanism drifts toward polynuclear growth, facets are lost since there is not enough time for the material to grow in the most energetically favorable configuration, being the quasi-spherical or even spherulitic particles more common (kinetic vs thermodynamic control).

As mentioned above, it was observed that the solvent played an important role during the formation of the crystalline structure of ZIF-8. As can be seen in Figure S1c, a dominance of water and methanol followed by DMF and other solvents was found. Therefore, the following analysis was restricted to the first two. Figure 2b showcases the relationship between the mechanisms described above (based on individual reports) and the general trends found in the literature. To this end, we have considered the nominal concentration reported by the authors. For both water- and methanol-based synthesis, the morphology of the particles evolves from faceted to quasi-spherical as the molar ratio increases, as seen by the shift on distribution medians. This can be also seen in Figure S4 where violin plots are shown. In the case of water, the vast majority of individual studies report poor faceted or quasi-spherical particles (one should always bear in mind that solvothermal conditions and modulator addition can turn this around).^58,68,69^ For methanol, there is a more evenly distributed number of reports regarding morphologies obtained, with one big difference though; the molar excess needed to achieve crystalline ZIF-8 particles is much lower than in the case of water-based synthesis, and only a very low excess, close to a stoichiometric 1:2 ratio, is enough to yield faceted particles. The absence of data points at higher molar ratios can be due to either unreported negative results or simply to the fact that those regions of the synthetic space remain unexplored.

Up to this point, a unified roadmap for nucleation and growth mechanisms was derived solely by analyzing individual reports considering supersaturation conditions. We have also shown how general trends extracted from the literature follow the proposed unified nucleation and growth map. It is now necessary to understand whether molar ratio is also determinant for the final shape and size of the ZIF-8 particles obtained, and to what extent the remaining synthetic variables have an impact on fit.^70,71^

### Molar Ratio as a Predictor of ZIF-8 Particles’ Features

**Figure 3:**
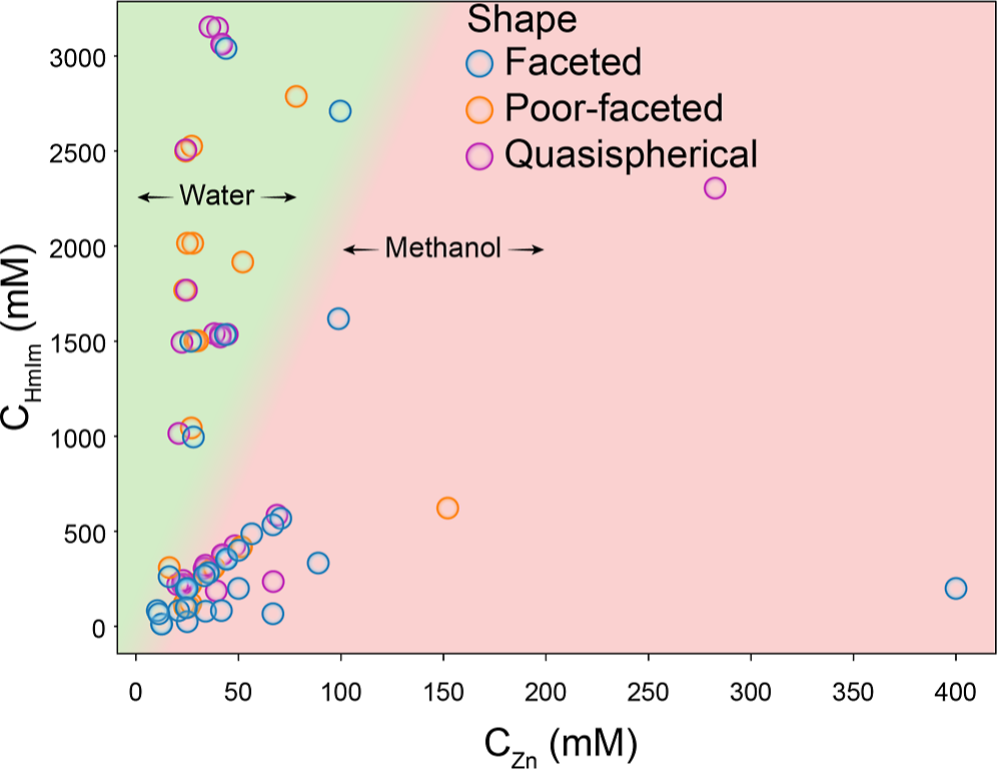
Relation between the morphology of reported ZIF-8 particles and both Zn^2+^ and HmIm final concentrations. Background gradient indicates the solvent employed at each region, being water in light green and methanol in light red. Please note that the gradient background has been made qualitatively and serves the sole purpose of guiding the eye.

If nominal concentrations (upon mixing) are considered instead of molar ratios, as shown in Figure 3, the partial exploration in the literature of the chemical space and the existence of regions for methanol- and water-based synthesis becomes clear. Methanol-based synthesis typically involves low concentrations of both precursors; on the contrary, water-based synthesis requires a large excess of HmIm and a low Zn concentration. Such required linker excess promotes the deprotonation of HmIm, which is necessary for the ZIF-8 formation.^67^ As previously mentioned, the solvent is involved not only in Hmim deprotonation and the solvation of the ions,^65^ but also acts as a structure director, allowing the framework to be built by bridging the pore structure through hydrogen-bond interactions during formation.^47,48,62,72−74^ However, if the solvent has a stronger ability for hydrogen-bond donation, then its interaction with the constitutive species of ZIF-8 and the intermediate complexes appearing during the synthesis will be too strong to allow the growth to continue. When this happens, a greater excess of HmIm is needed to boost nucleation and growth. The strong influence of the solvent was also reported for ZIF-67, an isostructural cobalt-based ZIF.^75^

A careful analysis of the data presented in Figure 3 shows no explicit trend; however, it can be seen that methanolic synthesis requires lower concentrations of precursors to achieve particles with a faceted morphology. Additionally, with an increase in the HmIm concentration, poor-faceted and quasi-spherical particles can be obtained (see Figure S5a for a disaggregated representation of overlapped data points). Aside from this, it is clear that the use of a nominal concentration of ZIF-8 precursors is not enough to describe the complexity of the system.

**Figure 4:**
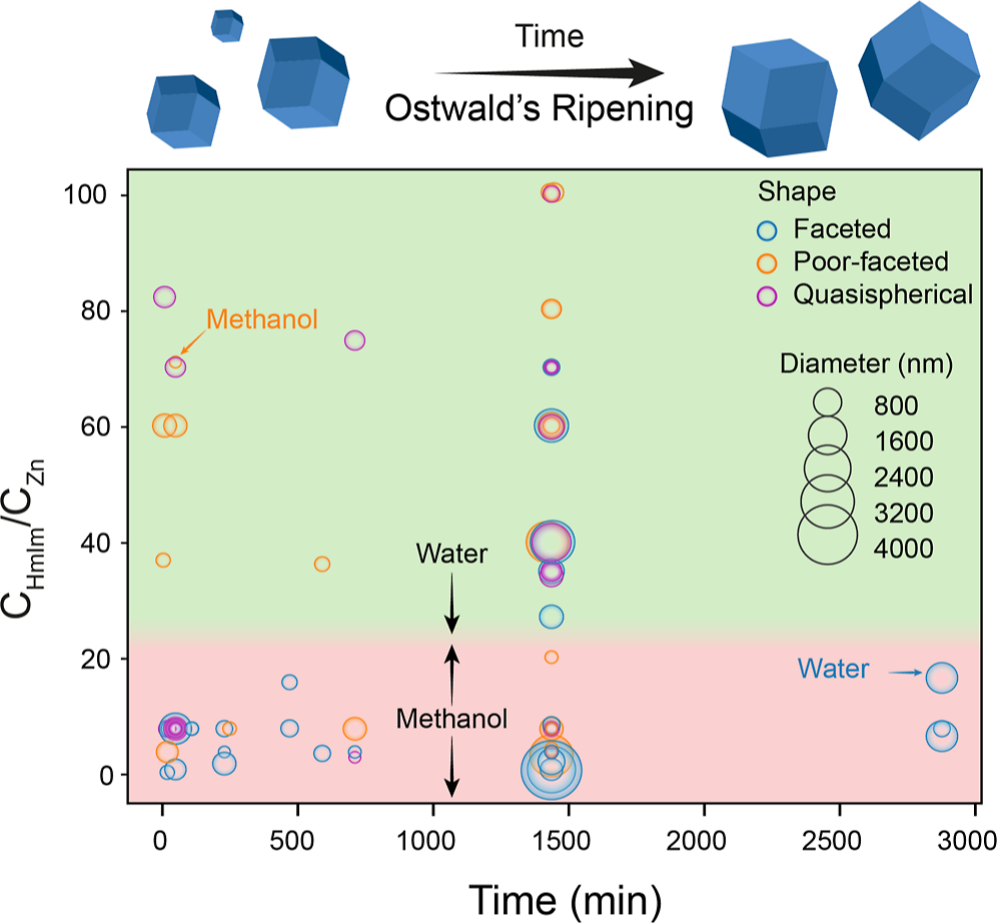
Time evolution of particle size (schematized by different circumferences) and morphology for different *C*_HmIm_/*C*_Zn_ molar ratios by Ostwald ripening. Background gradient indicates the solvent employed for each region, being water in light green and methanol in light red. Please note that the gradient background has been made qualitatively and serves the sole purpose of guiding the eye.

There are different mechanisms through which a particle can modify its size and shape. The well-known Ostwald ripening is a classic example of this. Here, smaller particles dissolve, and their constituents attach to the bigger particles, thus minimizing surface free energy. In the case of crystalline particles, the process also favors the densest crystalline planes (which for ZIF-8 favors the (110) plane, as discussed above) leading to rhombic-dodecahedral-shaped particles over the other faceted intermediates.^58^Figure 4 tracks the morphology and size of particles synthesized at different molar ratios over time. This plot aims to show a statistical trend of the behavior observed for Ostwald ripening; at low molar ratios, this should yield particles that increase their size as time elapses yet retaining their initial morphology. As can be seen in Figure 4, there is no clear dominance of faceted over quasi-spherical particles at longer times.

Several synthetic conditions, in both water and methanol, lead to the formation of ZIF-8 aggregates. Given the low number of entries in the data set involving aggregates, an in-depth analysis was not possible; however, it is worth noting that aggregates are formed in almost all the combinations reported. Aggregates can be found for both methanol- and water-based synthesis; with or without stirring; and involving particles falling into the three morphological categories (faceted, although fewer, poor-faceted, and quasi-spherical). It has been also reported that individual particles tend to have a relatively low polydispersity while aggregates differ in size.^49^ In addition, aggregation depends on other variables than excess ratio, probably related to kinetic aspects, such as active mixing through forced convection.^76^ This points toward a complex relationship among synthetic variables and the aggregation that needs to be systematically studied to improve our understanding of ZIF-8 (and MOFs, in general) aggregation.

From the above discussion, it becomes clear that the molar ratio and nominal concentrations of precursors allow for rationalization to some extent of the dominating mechanism for ZIF-8 formation, but extending the analysis toward the prediction of the size/morphologies of particles is not straightforward, and other variables or combinations of variables must be considered. As a first approach to understanding the available data in terms of the most frequently employed synthesis conditions and the correlation among each variable, distribution, and pair plots were obtained (Figures S7 and S8). Briefly, a pair plot (also known as scatterplot matrix) is a combination of individual scatter plots arranged in a way in which it is possible to analyze potential cross-correlations. The diagonal of a given pair plot shows a univariate distribution plot of the data (histograms). No clear trend was identified for the different synthesis variables and particle diameter/morphology explored, as might be anticipated from the complexity of the system. The analysis, however, showcases through the correlation factors, that all variables are interconnected and play a role in determining the final particle characteristics. To understand and extract useful information regarding the impact and relevance of synthetic variables involved in such highly multidimensional system, it becomes evident the need for more sophisticated methods like ML, applied to suitable databases.

One of the ZIF-8 main features, yet to be discussed, is its surface area. There has been attempts resorting to ML for structure optimization aiming to maximize MOF surface areas.^14,15,17,20,27^ Having this point in mind, we included in the collected database employed for the present study BET surface areas values reported. However, as shown in the SI file, no correlation was found between the particle’s size, morphology, and surface area; i.e., while synthetic conditions are crucial to determine the operating nucleation and growth mechanisms and the final particle size and morphology, they seem to have little effect on other functional properties, such as BET surface area. A possible rationalization of this fact might be that once the crystalline particle is formed, its surface area is well defined, and dispersion on the reported values can be ascribed to the methodology employed for the analysis of gas adsorption isotherms, as recently demonstrated in an extensive cross-laboratory collaborative research effort.^77^

### Determining the Impact of Synthetic Variables on the Final Particle Size through Machine Learning

**Figure 5:**
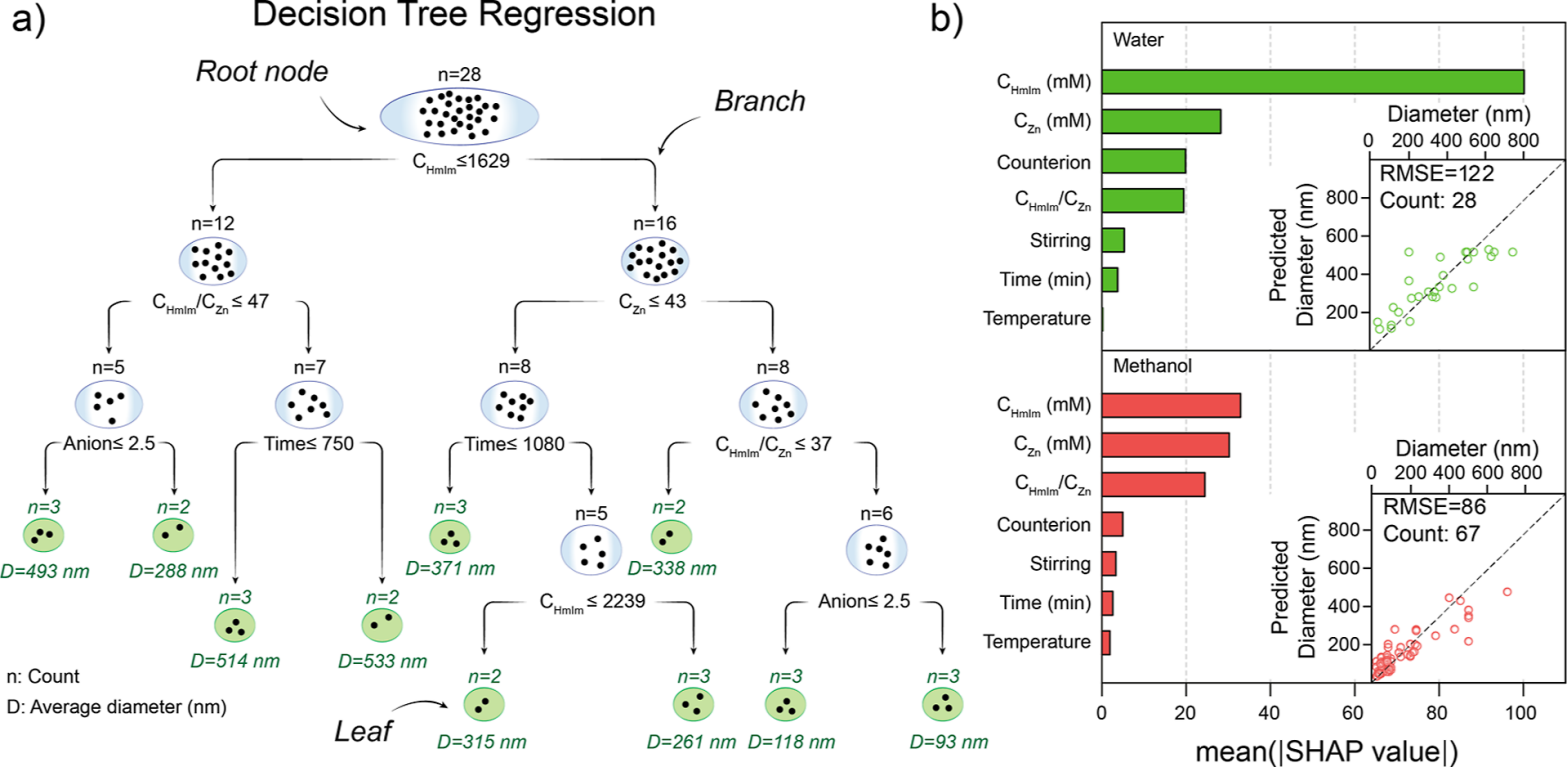
(a) Example of a single decision tree employed for Random Forest methodology. (b) Impact of individual variables on determining the final particle size for (top) water and (bottom) methanol. Insets: model fit for each solvent.

A model that can fit the data and explain a continuous variable, such as the size, is called a regression model. In ML, a basic and versatile model for making a regression are Decision Trees. Figure 5a shows an example of how a Decision Tree adjusts data coming from the aqueous-based synthesis of ZIF-8. The algorithm splits the starting data set (root node) based on a question or decision that reduces the variance in each new set and splits the data into two branches. Each of the new subsets is going to be split again by a different question, and the process will continue until the remaining data set cannot be split anymore, which constitutes the leaves of the tree. In this case, the minimum number of samples per leaf was set at 2 and maximum depth at 4 to limit overfitting.^78,79^ Finally, the average value per leaf was calculated as the outcome of each path. While individual decision trees can be interpreted in a relatively straightforward manner, the results might not be accurate, and there is still some risk of overfitting the data. To cope with this, a Random Forest methodology can be employed. Random Forest algorithms generate a final output by combining the individual output of multiple decision trees and 50 randomly created decision trees. By doing so overfitting is reduced, and a more accurate model is obtained. Model performance was assessed using leave-one-out cross-validation. In this approach, each data point serves as the testing set once, while the remaining data are used for training. This iterative process provides a robust estimate of the model’s generalization ability, which refers to its capacity to make accurate predictions on unseen data. The result indicates how well the model can predict new data. Once the model is built, it is then possible to know how much a given variable influences the final prediction, through the so-called SHAP value (SHapley Additive exPlanations).^80,81^ Herein, a random forest algorithm was applied to the data set, considering as relevant variables the concentrations, time, temperature, agitation (yes or no), and the counterion in zinc salt. For better contrast between methanol and water as a solvent, each one was modeled separately with a random forest.

Figure 5b shows mean SHAP values for the selected variables, discriminated according to the solvent employed. As previously mentioned, the model employed to analyze the initial data set from the literature will try to adjust the individual contributions of each variable over the main outcome, i.e., particle size. SHAP values are an indication of exactly that. In other words, SHAP values show which synthetic variable contributes the most to determining the final particle size. The insets in Figure 5b showcase the correlation between the prediction model and the input data. Although SHAP values are a great tool to understand the role of the variables on the overall response of the system, the correlation does not necessarily mean causality and, thus, the data set employed and its possible bias must be taken into account when analyzing the model output.

It is possible to exemplify this by considering the top panel of Figure 5b; the most decisive variable in controlling particle size for a water-based synthesis seems to be the HmIm concentration, while Zn concentration seems to be less important compared with HmIm. However, by looking at the pair plot diagonal (Figure S7b), it can be seen that the Zn concentration has not been widely explored in the literature, rather the opposite. This limits the validity range for the Zn SHAP value interpretation; it might be the case that Zn concentrations play a major role in controlling particle size, but there might not be enough reported data corresponding to water-based synthesis, thus affecting the ML analysis. Despite the above discussion, the precursor’s molar ratios have been explored up to 100 fold variation range, and although critical to establish the mechanism behind ZIF-8 nucleation and growth, it seems not to be a crucial parameter regarding particle size obtained. Although the influence of counterions in the Zn salt was scarcely explored (Figure S1b), there are a few systematic reports in the literature that suggest an actual effect on the size and morphology of particles.^70,73^ Bias on temperature’s role is also expected; by sorting out solvothermal synthesis conducted in hermetic vessels, room-temperature synthesis was mainly reported (with the associated variability of what temperature is considered room temperature in each case). The last point to be noticed is that time and stirring conditions seem not to play a significant role in controlling the final particle size, although it has been reported that stirring conditions influence aggregation and polydispersity.^76^

In the case of methanol-based synthesis, both HmIm and Zn concentrations seem to have equivalent relevance in determining the final particle size. When the distribution of reports for each variable is analyzed (Figure S7), it can be seen that, again, both concentrations have not been widely explored and that consequently, the explored molar ratio distribution is narrower than water-based synthesis. Distribution curves for stirring, counterions, and temperature conditions are also narrow, and showcase low SHAP values; reaction time, however, has been extensively varied for both solvents, and yet its impact on particle size also seems to be insignificant. This is in line with the previous discussion regarding Ostwald ripening.

**Figure 6:**
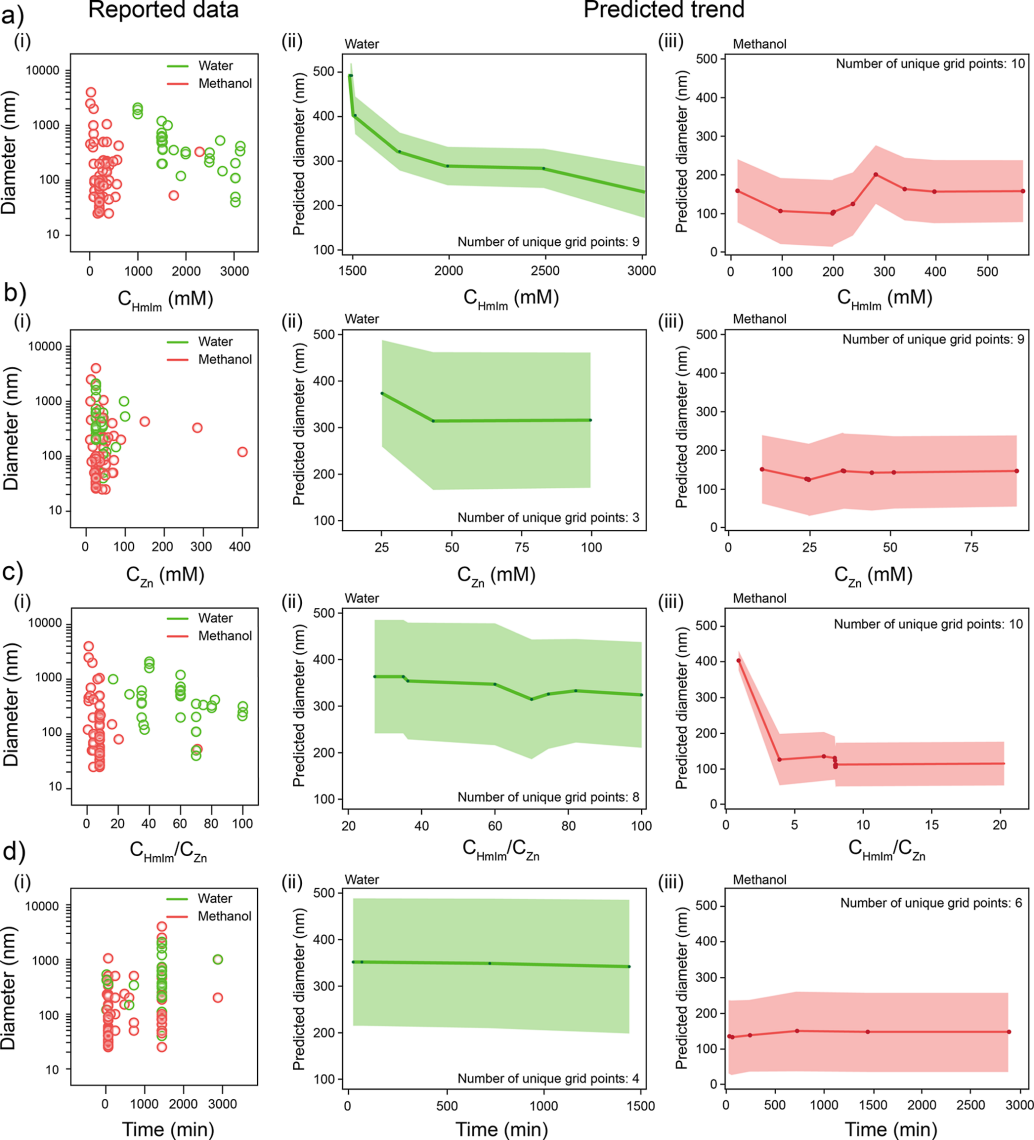
Reported individual data (left) and predicted response from partial dependence plots with a confidence interval (right), showcasing diameter variation upon changes in (a) Hmim concentration, (b) Zn concentration, (c) *C*_HmIm_/*C*_Zn_ molar ratio, and (d) synthesis time.

So far, the Random Forest approach has been useful in understanding which variables could have a significant impact on the final particles’ size, although with limitations due to the clustering of the data reported. It is possible now to analyze the reported data and obtain a predicted trend on how each one of the determined significant variables might influence particle size, while the remaining variables are kept constant, as summarized in Figure 6. This strategy is known as Partial Dependence Plot^82^ where the effect of a single feature is isolated on a model’s predictions by iterating through all possible values of the data set. For each value, a subset of the data is created, where all other features are constant. The model then predicts these subsets, and the predictions are averaged to account for inherent data variability and potential interactions with other features. The Partial Dependence Plot visualizes these average predictions for each value, revealing the overall trend of how some feature influences the model’s output.

To provide a rule-of-thumb for the analysis of the influence of synthetic variables in the growth of ZIF-8 particles, we extracted from our analysis the average predicted response with an associated confidence interval (excluding “flagrant” outliers, i.e., single data points in an otherwise clustered data set, as clearly seen in Figure 6a–d(i)). When considering, for example, water-based synthesis, as shown in Figure 6a(ii), the predicted trend for increasingly higher HmIm concentrations is in line with the above-proposed nucleation and growth mechanisms. At low concentrations, CNT holds, and growth proceeds via monomer (or even SBU) addition, allowing the particles to reach larger diameters. For higher concentrations of HmIm, the preferred pathway changes from a CNT to more complex mechanisms. Crystalline growth driving forces become more intense, leading to a faster generation of units that attach in an unordered fashion, at any available site, up to the point where diffusion is no longer the limiting step, and short-range phase segregation and internal reorganization take place. Considering the dual role of HmIm, (being both a bridging unit and a terminating agent, thus limiting particle growth), it is then expected that particles become smaller when a large excess of the linker is employed.^45^ When resorting to methanol-based synthesis, as seen in Figure 6a(iii), the behavior is the same as that observed for water-based synthesis and features an increase followed by a constant plateau. The change in trend takes place after 250 mM concentration, which is above the mode of the distribution (see Figure S6b), suggesting that there might not be enough data in that region to provide an appropriate data set for deriving a model.

Analyzing the effect of Zn concentration is less straightforward since experimental reports with a systematic exploration of this variable are lacking, as becomes evident from Figure S6b. For both cases, water- and methanol-based synthesis, the mode of Zn concentration distribution is 50 mM (with some higher values explored in water, at 100 mM). Together with the removal of outliers and considering the confidence intervals, this translates into a rather constant trend in the case of methanol for concentrations above the mode and a slightly decreasing trend in the predicted diameter with increasing Zn concentration for water-based synthesis. Given the concentration range, CNT also holds in this case; an increase in Zn concentration would lead to a larger number of nuclei being formed in the initial stages of the synthetic process, thus leading to smaller particles. This can also be analyzed by considering the surface energy of the ZIF-8 phase in water. It has been reported that the relative decrease of HmIm concentration leads to a ZIF-8 phase with a higher −OH surface termination, which increases surface energy and presents a high surface barrier toward crystallization.^61^ One of the reasons behind it is the energy associated with water coordination on Zn sites (−153 kJ·mol^–1^) being comparable with the energy of HmIm coordination on free Zn sites (−191 kJ·mol^–1^).^61,63^ Further systematic exploration of the chemical space might shed light on the impact of the Zn concentration in both water- and methanol-based synthesis.

Panel (c) in Figure 6 shows the predicted trend upon variation of the Zn/HmIm molar ratio, which is then a convolution of the above-discussed trends. Considering the narrow distribution of Zn concentrations explored in the literature, the effect of modifying the molar ratio could be explained in the same terms that the predicted trend for HmIm concentration changes, and although the molar ratio has been proposed in the literature as a tool to control particle size, based on our analysis it will rather set the nucleation and growth mechanism, while the final particle size will be determined by other variables. Finally, as seen in Figure 6d, elapsed synthesis time does not seem to have a significant impact on determining the final particle size, in line with the previously discussed lack-of-clear trend observed in Figure 4. It is important to note that this would not mean that Ostwald́s ripening mechanism is not operating; it rather suggests that the final particle size is mainly dictated by the other synthetic variables.

**Figure 7:**
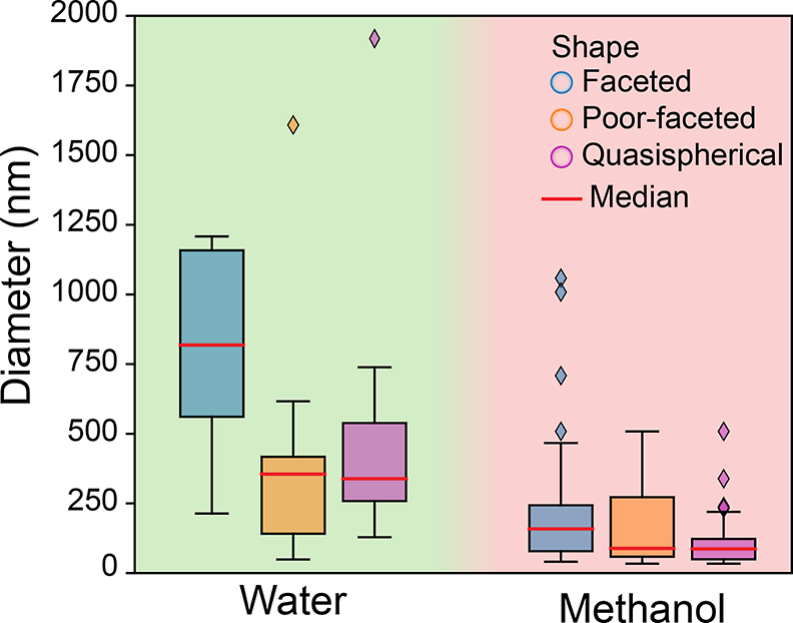
Diameter distribution for the different particles reported in the literature, classified by shape (faceted, poor-faceted, quasi-spherical) grouped by solvent.

Observations discussed above suggest that conditions leading to faceted particles correspond to low supersaturation regimes, as introduced in Figure 2b, i.e., high Zn concentrations and/or low HmIm concentrations, and with larger diameters than poor-faceted particles; and that the latter will be larger than quasi-spherical particles. In terms of the operating mechanism, as supersaturation increases, which means either decreasing Zn concentration and/or increasing HmIm content, then monomer or SBU’s addition mechanism will transition to polynuclear growth, internal reorganization, or even aggregative growth, and due to the terminating effect of HmIm large excess, particles will become less faceted and smaller. This trend is visible in Figure 7, where a correlation between the diameter and the morphology of the particles for each solvent (i.e., water and methanol) is presented. The median diameter value decreases when losing the faceted condition for both water- and methanol-based synthesis, thus supporting the previously discussed hypothesis. These results aim to understand the main trends found in literature, thus providing a general understanding of the mechanisms operating to obtain particles with a given set of features.

## Conclusions

The findings presented in this paper represent a significant step forward in the understanding of the nucleation and growth mechanisms underlying the formation of Zeolitic Imidazolate Framework-8 (ZIF-8) nanoparticles. Employing ML applied to an ad-hoc dataset generated by hand-picking reports published since 2006, we have been able to extract meaningful insights and provide a unified roadmap for the growth of this particular MOF, and can be used as a guide for future research.

One of the key findings of this work is the importance of the molar ratio between Zn and 2-methylimidazole HmIm in determining the operative mechanism for ZIF-8 formation, which can transition from growth through addition of single ions to the formation of an amorphous clusters, and later to internal reorganization. Additionally, we have demonstrated that no single synthetic variable alone can fully control nor be employed to predict all aspects of ZIF-8 particle size and morphology, and that it is only through analyzing the complex interplay between these variables that a true understanding of the growth process can be obtained.

Moreover, by using ML protocols, we have been able to determine the specific influence of each synthetic variable on the median particle size, shedding light into the role of each single parameter controlling the final product properties. This is an important step forward in the development of MOFs with tailored properties, which have significant implications for a wide range of potential applications. However, we also acknowledge that this work represents only a starting point in the quest for a complete understanding of the nucleation and growth mechanisms of ZIF-8, and there is still much work to be done to fully explore the chemical space of these materials. On a general note, we believe it is imperative to set standards for reporting synthesis results that allow systematization and, more importantly and perhaps ”contracultural”, to include “negative or non-desired” results in our reports, which will allow depicting the complete chemical space. We believe that the results presented here will be of significant utility to the scientific community, serving as a guide for future research on ZIF-8, with a methodology that can be easily extrapolated to other MOFs and related materials.

## Supporting Information Available

Information about database construction, general introduction to nucleation and growth theories, disaggregated concentration plots, pair plots, and BET surface area vs particle diameter for each morphology (PDF)
