# Supplementary material for: Unified Roadmap for ZIF-8 Nucleation and Growth: Machine Learning Analysis of Synthetic Variables and Their Impact on Particle Size and Morphology
Source: Chem Mater. 2024 May 20;36(11):5814–25. doi: 10.1021/acs.chemmater.4c01069 (PMC11171283; doi:10.1021/acs.chemmater.4c01069)
Supplement: Supplementary file 1 — cm4c01069_si_001.pdf [file cm4c01069_si_001.pdf]

Supporting Information: Unified roadmap for ZIF-8 Nucleation and Growth: Machine Learning Analysis of Synthetic Variables and their Impact on Particle Size and Morphology

Juan A. Allegretto,[*,†,‡,¶] Diego Onna,[*,§,‖,†] Sara A. Bilmes,[§,‖] Omar Azzaroni,[¶] and Matías Rafti[*,¶]

†*Joint first authors*

‡*Laboratory for Life Sciences and Technology (LiST), Faculty of Medicine and Dentistry, Danube Private University, 3500 Krems, Austria.*

¶*Instituto de Investigaciones Fisicoquímicas Teóricas y Aplicadas (INIFTA), Departamento de Química, Facultad de Ciencias Exactas, Universidad Nacional de La Plata, CONICET, CC 16 Suc. 4, La Plata B1904DPI, Argentina*

§*Instituto de Química Física de los Materiales, Medio Ambiente y Energía (INQUIMAE), CONICET-Universidad de Buenos Aires, Buenos Aires, Argentina.*

‖*Departamento de Química Inorgánica, Analítica y Química Física, Facultad de Ciencias Exactas y Naturales, Universidad de Buenos Aires, Buenos Aires, Argentina.*

E-mail: juan.allegretto@dp-uni.ac.at; diego.onna@qi.fcen.uba.ar; mrafti@quimica.unlp.edu.ar

Methodology and database generation

As schematized in Figure S1, the data set for this work was generated by collecting research articles from Scopus using different combinations of keywords: *ZIF-8*, *Zeolitic*,*Framework*, and *MOF*, giving a total of 3440 entries. After this, duplicates and Review articles were identified and deleted, for a final 2248 entries. A total of 165 articles were hand-picked by title+abstract analysis, starting with the highest cite score.

One by one, the following parameters were extracted: Zinc source and the amount employed for the synthesis in mmol (milli-moles); 2-methylimidazole in mmol (hereinafter $HmIm$); solvent and quantity employed (in mmol). Then, temperature, time, and stirring conditions during synthesis; quiescent synthesis, (parametrized as 0) was discriminated from stirring conditions; stirring used just at the beginning (parametrized as 1) or during the entire synthetic time (parametrized as 2) was also classified. Finally, it was also registered if the article was systematic (39.24 %); i.e., whether or not the authors explored systematically the effects of any variable over the ZIF-8 characteristics. After these, features of the obtained materials were extracted. Given that not every work analyzed simultaneously all textural characteristics (size, crystallinity, morphology, porosity, etc.) from the synthesized particles, only particle size, morphology, and porosity (in terms of BET area) were extracted. Particle size was discriminated according to the technique employed; DLS, TEM, SEM, SAXS, or Crystalline size (by Scherrer equation). To establish a common base of comparison, only TEM/SEM information was employed in the analysis. From the initial 165 articles, only 79 reported any information about particle sizes (47.88 %) and were included in the curated database, representing a total of 254 entries (i.e., 254 individual sets of synthetic conditions with information about the resulting particles analysis). The data set is openly available in Zenodo.[S1]

For those works where SEM/TEM images were available but no information about particle size distribution was reported by authors (13.78 %), ImageJ software was employed to extract the information from the reported images, assuming they were representative of the

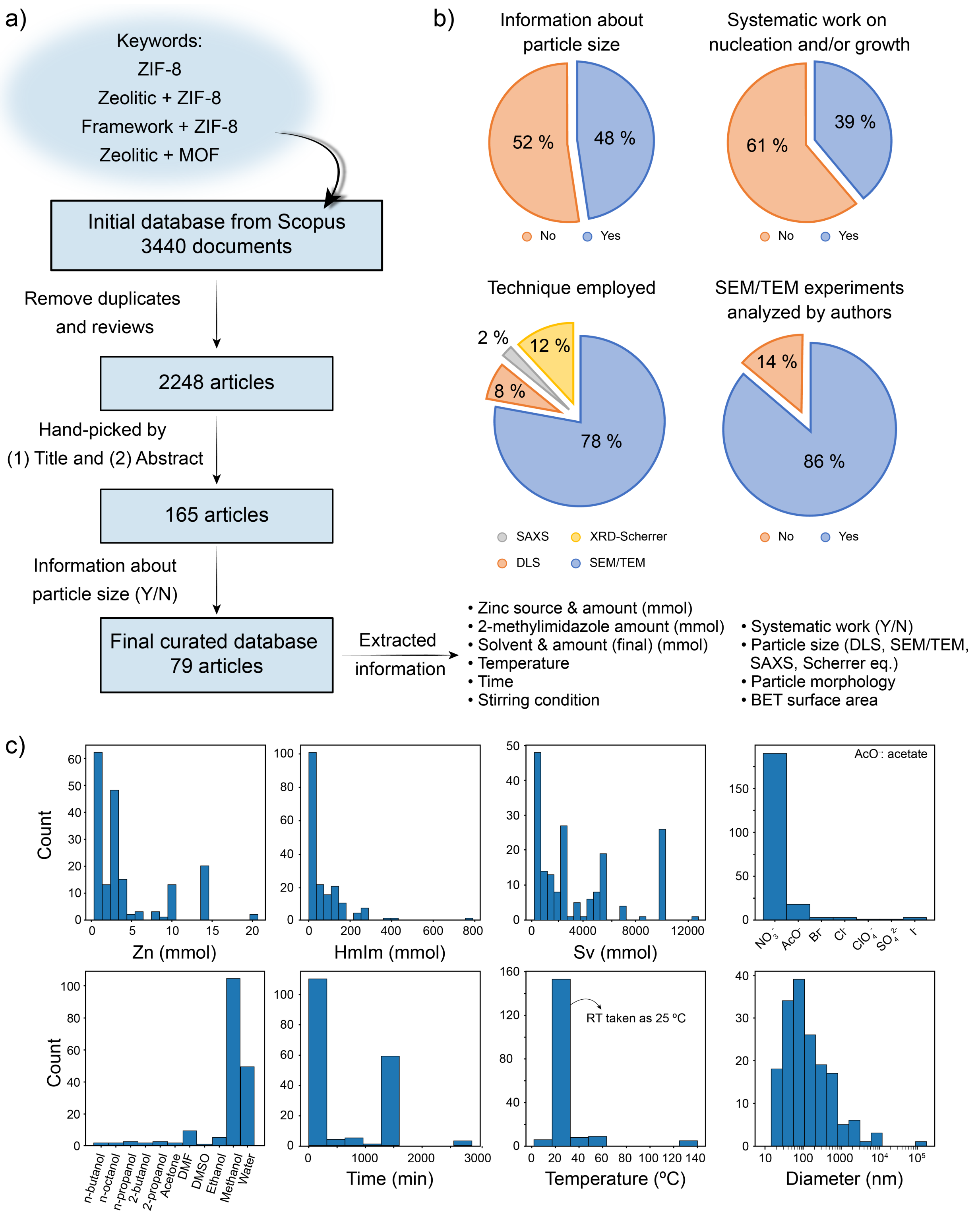

Figure S1: Schematic representation of database construction, curation, and extracted data.

entire sample. The outcome of this entire process is summarized in Figure S1a-b.

Figure S1c shows histograms of the individual parameters extracted. The curated database (i.e., the database composed of 79 documents, with a total of 254 synthetic conditions) is presented as supplementary material.

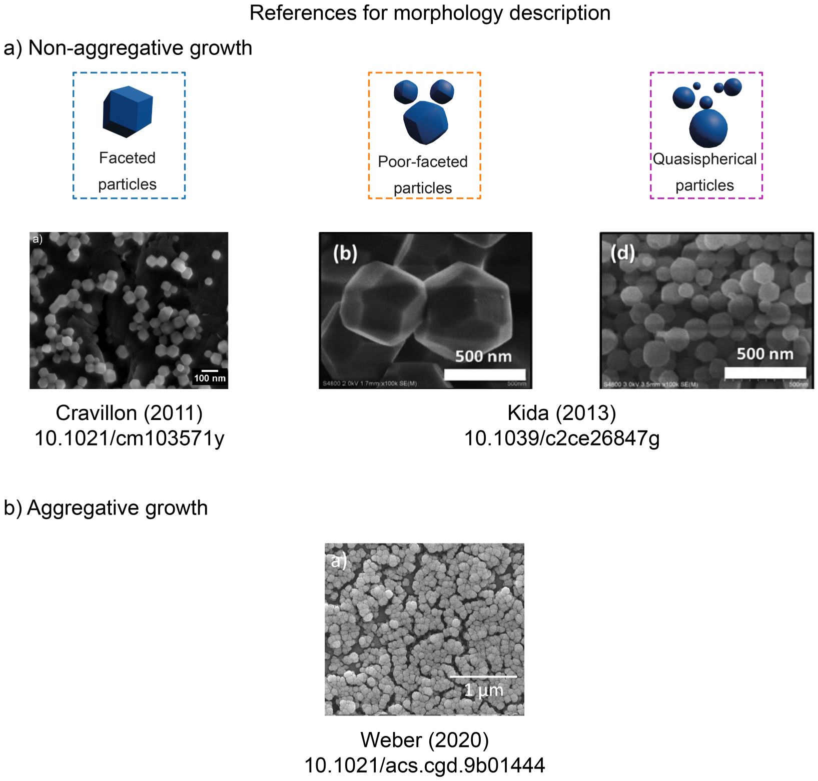

Figure S2: Reference for morphology assignment of ZIF-8 particles as (a) Faceted particles. Adapted with permission from ref.[S2] Copyright 2011 American Chemical Society; Poor-faceted and Quasispherical particles. Reproduced from ref.[S3] with permission from the Royal Society of Chemistry. (b) Aggregative growth example. Adapted with permission from ref.[S4] Copyright 2020 American Chemical Society.

To describe the morphology of the particles, three categories were applied, namely: (i) faceted particles (particles with well-defined edges, with rhombic dodecahedral shape or truncated rhombic dodecahedral); (ii) poor-faceted particles (particles with visible edges yet poorly defined); (iii) quasispherical particles (round particles with no visible edges). Additionally, particles forming aggregates were also classified and analyzed separately. All these categories are exemplified in Figure S2.

Table S1: References for Figure 1 in the main manuscript

Legend	DOI	Ref
Bohme (2013)	10.1021/la401471g	S5
Bustamante (2014)	10.1016/j.jcis.2014.03.014	S6
Chang (2010)	10.1021/ja1058229	S7
Cheng (2020)	10.1016/j.matchemphys.2020.122869	S8
Cravillon (2009)	10.1021/cm900166h	S9
Cravillon (2011b)	10.1002/anie.201102071	S10
He (2014)	10.1016/j.micromeso.2013.10.003	S11
He (2019)	10.1039/c8sc03520b	S12
Jayaramulu (2015)	10.1002/anie.201507692	S13
Jian (2015)	10.1039/C5RA04033G	S14
Jiang (2017)	10.1021/acsami.7b04432	S15
Khan (2014)	10.1016/j.jhazmat.2014.03.047	S16
Kida (2013)	10.1039/c2ce26847g	S3
Lai (2014)	10.1080/02726351.2014.920445	S17
Lai (2017)	10.1021/acscatal.6b02966	S18
Lee (2015a)	10.1016/j.cej.2015.02.094	S19
Lee (2015b)	10.1021/acs.jpcc.5b01519	S20
Li (2014)	10.1021/jp508381m	S21
Li (2017)	10.1039/c6nr08987a	S22
Lu (2012)	10.1038/nchem.1272	S23
Pan (2011)	10.1039/c0cc05002d	S24
Saghir (2021)	10.1016/j.materresbull.2021.111372	S25
Schejn (2014)	10.1039/C3CE42485E	S26
Shen (2018)	10.1126/science.aao3403	S27
Tran (2011)	10.1021/cs1000625	S28
Tsai (2016)	10.1016/j.micromeso.2015.08.041	S29
Venna (2010)	10.1021/ja109268m	S30
Wang (2018b)	10.1002/adfm.201802596	S31
Weber (2020)	10.1021/acs.cgd.9b01444	S4
Wu (2014)	10.1039/c3nr04390h	S32
Zhang (2012)	10.1021/jz300855a	S33
Zhang (2013)	10.1021/jz402019d	S34
Zhang (2014a)	10.1021/ja5084128	S35
Zheng (2016)	10.1021/jacs.5b11720	S36
Zhou (2017)	10.1039/C6TA07860E	S37
Zhu (2013)	10.1016/j.catcom.2012.12.003	S38
Zhu (2017)	10.1038/nmat4852	S39

Overview on nucleation and growth mechanisms

The following is a compendium of different sources, where nucleation and growth are addressed from different points of view.[S40–S44] The goal of this section is to link them together, highlighting the differences between them and underlying the driving force of each of them and thus providing a minimal common ground for the discussion throughout this manuscript.

Nucleation is considered as the initial process for the formation of a crystal and is typically explained in terms of the *supersaturation*. The condition of chemical equilibrium in a homogeneous system is given by the equality of chemical potentials of every chemical species present. In a heterogeneous system, the role of chemical potentials is the driving force in all phenomena dealing with molecular transport, from the phase with higher chemical potential μ' to the phase with the lower one, μ''. The resulting *chemical work* gained by the molecular transport, is typically called *supersaturation*, and it can be written as $\Delta\mu = \mu' - \mu''$. In ideal solutions, supersaturation can be written in terms of concentrations, as given by:

$$\Delta\mu = k_B T ln\left(\frac{c}{c_0}\right) \tag{1}$$

were k_B is the Boltzmann constant, T is the absolute temperature, and c and c_0 are the concentration of a supersaturated and saturated solutions. Nucleation and growth models can be grouped into classic and non-classic mechanisms, each of them taking place under different supersaturation conditions:

For nucleation mechanisms:

- Classic Nucleation Theory

  1. Homogeneous Nucleation
  2. Secondary and Heterogeneous Nucleation

- Zeolite Nucleation mechanisms

  1. Monomer

2. Secondary Building Unit
3. Nanoslab

As for growth mechanisms:

- Classic Model
  1. Thermodynamic approach
  2. Kinetic approach
     (a) Diffusion-limited
     (b) Surface reaction-limited, which includes (i) Mononuclear growth. (ii) Polynuclear growth (iii) Adhesive or spherulitic growth.
- Non-classic growth mechanisms
  1. Ostwald's Sept Rule
  2. Aggregative growth

Under Classic Nucleation Theory (CNT), the formation of crystal nuclei occurs after the constituent atoms and/or molecules of the crystal have come together and positioned into a fixed lattice. Under supersaturation conditions, crystallization is thermodynamically favored, and nucleation of a particle with radius r generates a change in free energy ΔG_c, associated with an interfacial free energy per unit area ΔG_a and the energy required to form the bulk of the particle, in terms of volume unit ΔG_v:

$$\begin{aligned}\Delta G_c &= \Delta G_a + \Delta G_v \\ &= 4\pi r^2\gamma - \frac{4}{3}\pi r^3 \Delta\mu\end{aligned} \tag{2}$$

where γ is the surface tension of the new particle. CNT predicts the existence of a critical radius r^*:

$$\frac{d\Delta G_c}{dr} = r^* = \frac{-2\gamma}{\Delta\mu} \tag{3}$$

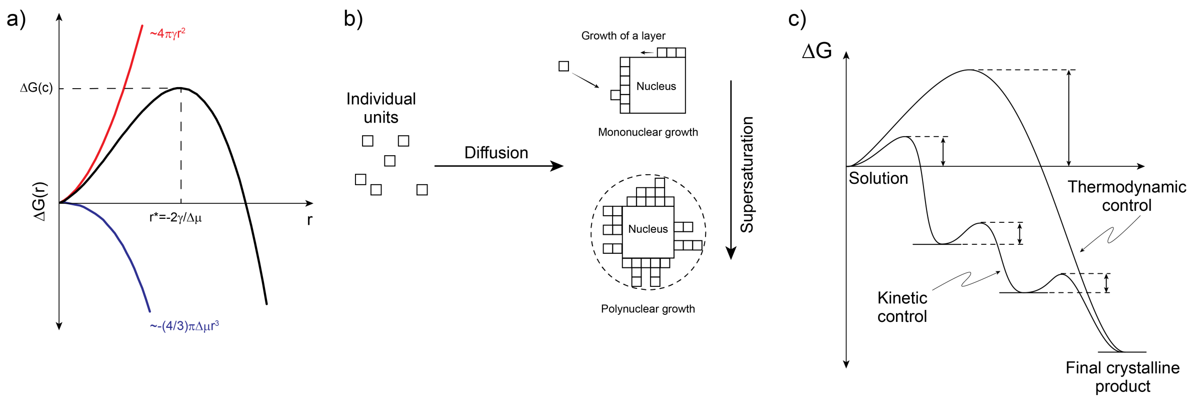

Figure S3: Schematics of the (a) formation of nuclei with critical radii according to Classical Nucleation Theory. (b) Diffusion-limited and attachment-limited growth processes. (c) Ostwald's Step Rule.

If a nucleus radius is smaller than r^*, it dissolves; nuclei that reach r^* become stable and continue to grow by a given mechanism. This process is schematized in S3a. The previous considerations are based on a free-standing particle growing in a homogeneous phase. When a crystal nucleates on preexisting nuclei of the same material, nucleation is called *Secondary* nucleation and is considered a different phenomenon than growth (see later). When a crystal is formed on a foreign surface, it is called *heterogeneous* nucleation. Also, CNT implicitly assumes that any nucleation leads directly to the formation of the most thermodynamically stable phase, with no amorphous intermediates nor polymorphous, which might not be true for every system, such as Zeolites.

It is commonly accepted that nucleation in Zeolites is based on the generation/breaking of $Si/Al - O$ bonds. The differences between models are how this happens and which intermediates are involved. In the zeolites monomer model, for example, there is an initial formation of an amorphous gel-like phase that evolves by local-restructuring into a crystalline phase. It is important to separate this model from the CNT monomer model, which predicts that nucleation takes place at the interface between a homogeneous solution and the growing particle, by the addition of individual units. Rather than individual units, nucleation can also take place by the addition of the so-called *Secondary Building Units* (SBU), which are the

minimum structural units of the crystal. Zeolite formation by the SBU model also involves the formation of the amorphous gel-like phase, but here it acts as either a reservoir of SBUs or as a heterogeneous nucleation site. The nuclei grow then, maintaining the final crystalline phase, without any intermediate phase, like in CNT nucleation. CNT also considers the process of an SBU attaching homogeneously on a surface, following the crystalline structure of the most thermodynamically stable phase.

Somewhere between the accepted limit of nucleation and growth, it falls under the proposed Nanoslab model for zeolites; here, crystalline particles are formed by the internal reorganization of pre-existing nanoparticles. Nanoslab resembles an aggregative process, where nanoparticles aggregate in an oriented fashion and the final crystal is defined as the aggregate of monocrystalline domains with well-defined sizes and orientations.

Once stable nuclei are formed by any of the above-discussed mechanisms, those nuclei must grow. Classical growth models consider the addition of a given entity to the growing nuclei, and how this addition is taking place. From the thermodynamic point of view, crystal growth is favorable at supersaturation conditions and proceeds to yield morphologies that minimize the total Gibbs surface free energy. From the kinetic point of view, individual units must reach the surface of the growing crystal and then attach to it. According to the limiting process, crystalline growth will be dominated either by diffusion or by the interaction between the individual units and the crystal's surface, as summarized in S3b.

When the limiting process is not the diffusion but the attachment on the crystal's surface, there are mainly three different scenarios, according to the supersaturation degree. At low supersaturations, the resulting crystalline surface is smooth. Individual units adsorb onto energetically favorable points, what is typically known as *mononuclear* regime (different from zeolites' mononuclear mechanism). Here, each "layer" has enough time to complete its growth before the next layer starts to grow. Because of this, particles with well-defined edges are obtained, favoring the most dense crystalline planes. For moderate-supersaturated regimes, small units can adsorb on steps or kink sites by "birth and spread" mechanisms.

Surface attachment here is so fast that atomic layers are not complete before the next one starts to grow, which is known as *polynuclear growth.* However, at high supersaturation regimes, crystalline growth driving forces increase, and so units attach at the surface at any available site. This mechanism is typically known as adhesive growth and yields crystals with fractal, dendritic, or spherulitic shapes.

The classical mechanisms described so far, consider the growth as an amplification process, in which a stable nucleus increases its size without any structural change, either in bulk or in the crystal's surface. Classical models also assume that during growth, no change in crystalline phase will take place; and that the growth proceeds by the addition of small and individual units. However, under a given condition these assumptions might not hold, specially if the system is under kinetic control. One of the models that accounts for non-classical growth is Ostwald's Step Rule. Ostwald's rule is typically seen for polymorphic materials, where molecules can adopt different arrangements, either amorphous or crystalline, each of which will have a different energetic barrier, as shown in S3c. Under these conditions, crystallization of the same final crystal can take place by successive precipitations; the phase with the lower energetic barrier is the one that appears first, instead of the thermodynamically favorable phase. Later, this phase will suffer a transformation, for instance, dissolution-recrystallization or solid-phase reorganizations. The latter requires the initial and final phases to be structurally related and to have identical construction units. These mechanisms are highly sensitive to reaction parameters, such as the solvent, temperature, modulators, etc.

Finally, crystals can grow by an aggregative mechanism. Here, pre-formed construction units aggregate to form a crystal. This aggregation is typically oriented and it is influenced by the morphology of the original particles. The Nanoslab mechanism proposed for Zeolites nucleation is sometimes considered an aggregative growth mechanism.

It is important to highlight at this point the following. Across the manuscript, we have employed the word "particle" understanding that a particle can be amorphous, polycrys-

talline, or even a single crystal. Then, if we consider the particle formation (nucleation and growth), this can take place either with the utmost control thus ultimately leading to the formation of single crystals, where no grain boundaries are present and the entire extent of the solid (whichever it´s dimensioned) can be considered as a continuous repetition of unit cells in the three dimensions; or by a kinetic control, where polycrystalline and irregular particles are obtained. Between these two extremes, we can encounter all kinds of mechanisms, which is what we aim to summarize and show at the beginning of our manuscript. Where to set the limit between a single crystal and a nanoparticle with a high degree of crystallinity is, to the best of our knowledge, a matter of discussion today. Due to the limited data reported across the reviewed manuscripts, to discern whether or not the particles are indeed single crystals when the authors did not provide a deep analysis in those lines is far from trivial, and we can only attempt to classify those particles as either crystalline or not (first) and then obtain some insight based on the morphology of the particle, understanding that a faceted particle with well-defined facets will be closer to the single crystal case. At this point, it is necessary to include the ongoing discussion in the community on which nucleation and growth mechanisms operate for the formation of ZIF-8. As we tried to postulate at the beginning of our manuscript, the individual reports are only proving a limited section of the chemical space that leads to the obtention of ZIF-8, and it is only until those reports are discussed together that we can have a clearer vision of the processes taking place.

Violin plots

Figure 2b in the main manuscript showcases the distribution of ZIF-8 particles with the different morphologies, based on the concentration ratio C_{HmIm}/C_{Zn}, separated by water- and methanol-based synthesis. This graph gives a quick view of the main trends and concentration ranges explored in each case, by including all the reported values included in our dataset after curation, except for flagrant outliers, individually represented. For analyzing each subcase (i.e., water- and methanol-based synthesis), Figure S4 presents the data in a shorter range, excluding outliers. Here, it can be easily seen by analyzing the median of the distribution, that as the molar ratio increases, the obtained particles shift from faceted to poor-faceted and ultimately quasispherical morphologies, thus supporting the progression of nucleation and growth mechanisms proposed.

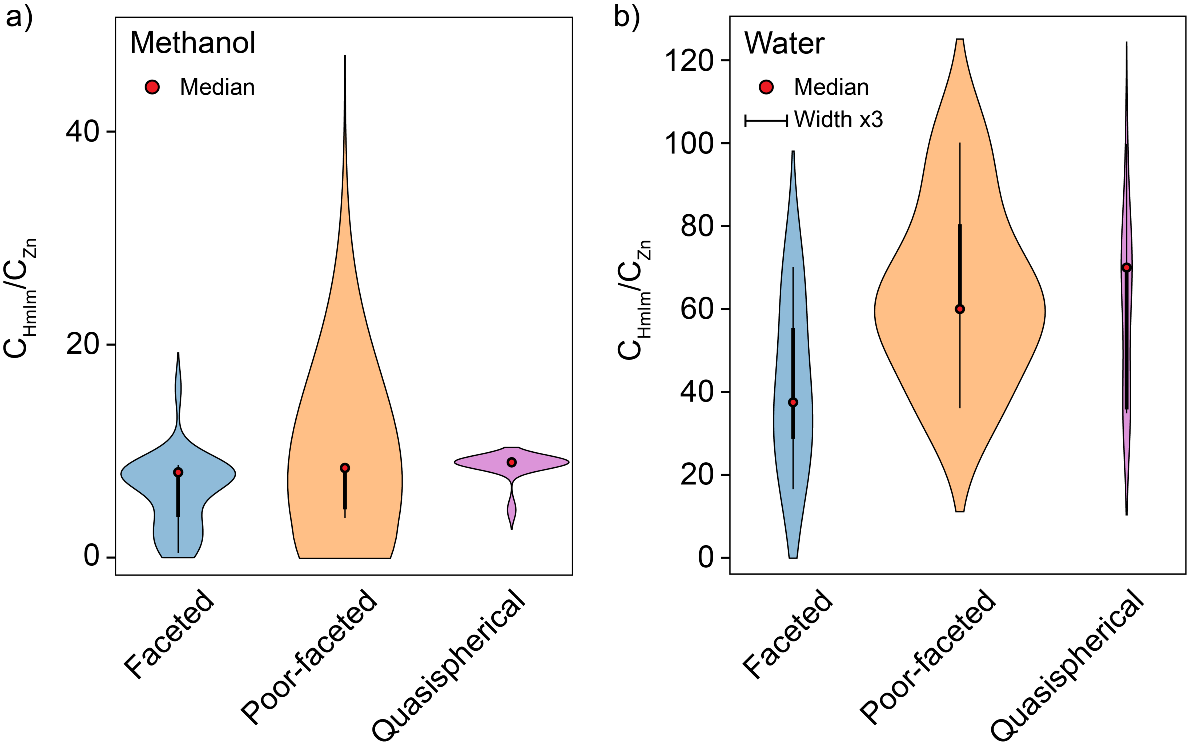

Figure S4: Violin plot distribution of ZIF-8 particles with faceted, poor-faceted, and quasi-spherical morphologies for (a) methanol- and (b) water-based synthesis. The width of plots in (b) is magnified x3 for better showcasing of the distribution.

Concentration plots

As described in the main manuscript, several synthetic conditions are widely employed. Because of this, overlapped data points were found, particularly for plots related to the nominal concentration of the employed reactants. This can be visualized in Figure S5, where data points were displaced by hand. **Please note that displacement of data points in the highlighted areas was done arbitrarily and holds no correlation with the actual concentration, serving the sole purpose of visualizing the otherwise overlapped data points.**

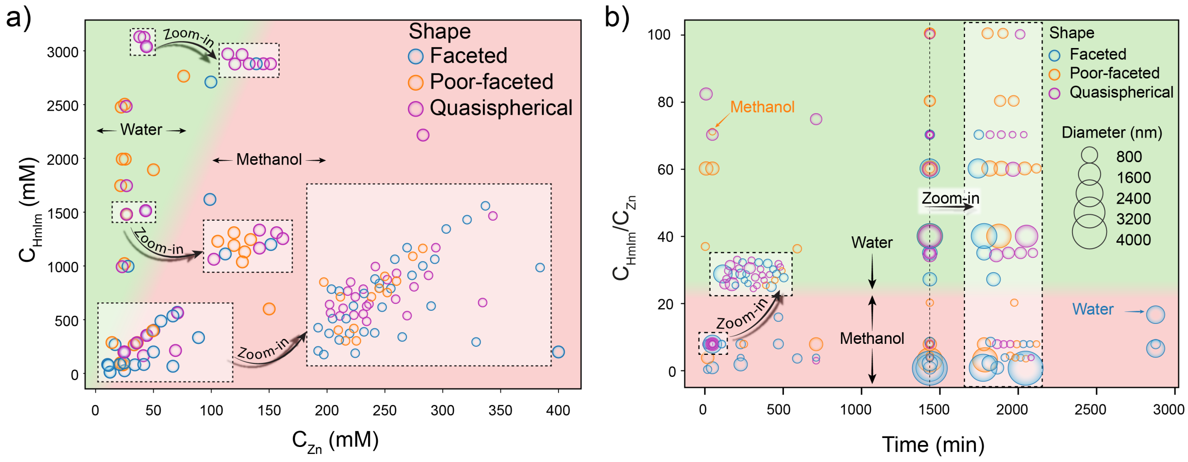

Figure S5: Plots for (a) particle morphology according to $HmIm$ and Zn concentrations and (b) time evolution of particles with different morphologies, were overlapped datapoints were displaced by hand.

Additionally, Figure S6 is presented where individual data points are replaced by isolines representing concentrations where it is possible to find particles with a certain shape. Notice that as in the previous visualisations there is a lot of overlap between possible domains.

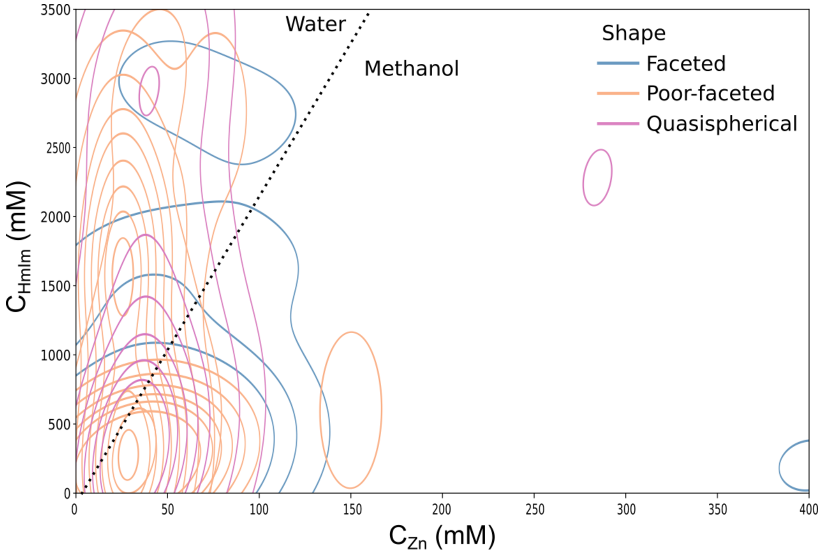

Figure S6: Contour plot for particle morphology according to $HmIm$ and Zn concentrations.

Pair Plots

Also known as scatter-plot matrix, pair plots are a square arrangement of scatter-plots, from which different pairs of variables can be cross-examined, and are useful tools to reveal and understand trends between different variables. The diagonal of the pair plot, results in a histogram of the variable.

Figure S7a shows a pair plot constructed with different synthetic variables, such as Zn and $HmIm$ concentration, their molar ratio, time, and final diameter of the particles. The upper half of the pair plot, classifies the data according to the morphology of the particle, while the bottom part of the plot, classifies the data according to the solvent employed. Both halves are mirror images. To take advantage of this, two pair plots are shown at once, where the upper part classifies the data points based on the particle shape, while the bottom part classifies them by solvent employed during the synthesis. The diagonal of the pair plots was extracted and is presented in S7b for clarity.

Figure S8 shows the same pair plot arrangement, yet classified according to the counterion employed. As can be seen, the synthesis reported employs mainly nitrate as counterion, followed by some reports using acetate and then other counterions to a very low extent.

a)

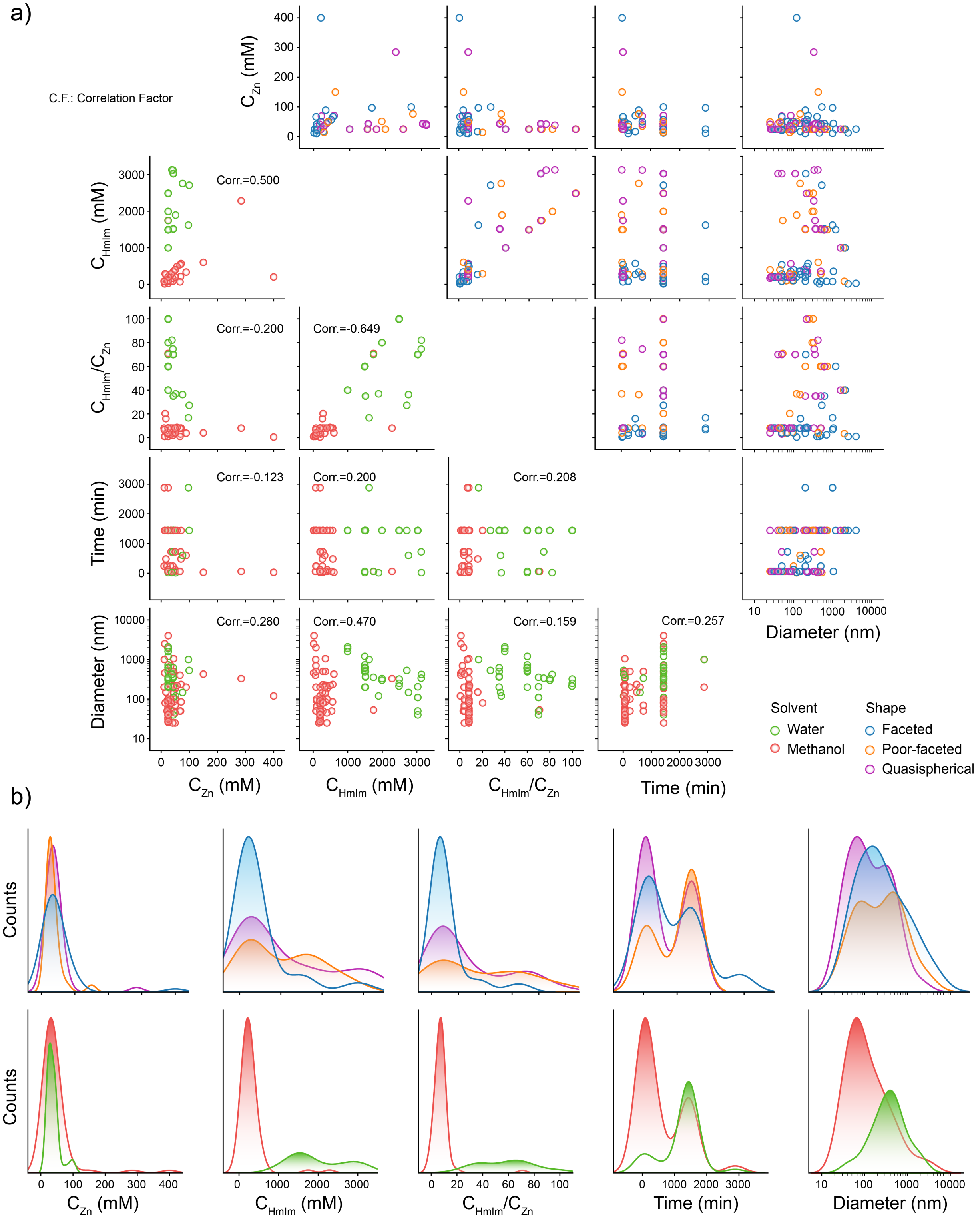

Figure S7: (a) Pair plot for different synthetic variables, classify according to particle morphology (upper half) and solvent (bottom half). (b) The pair plot's diagonal is extracted for clarity.

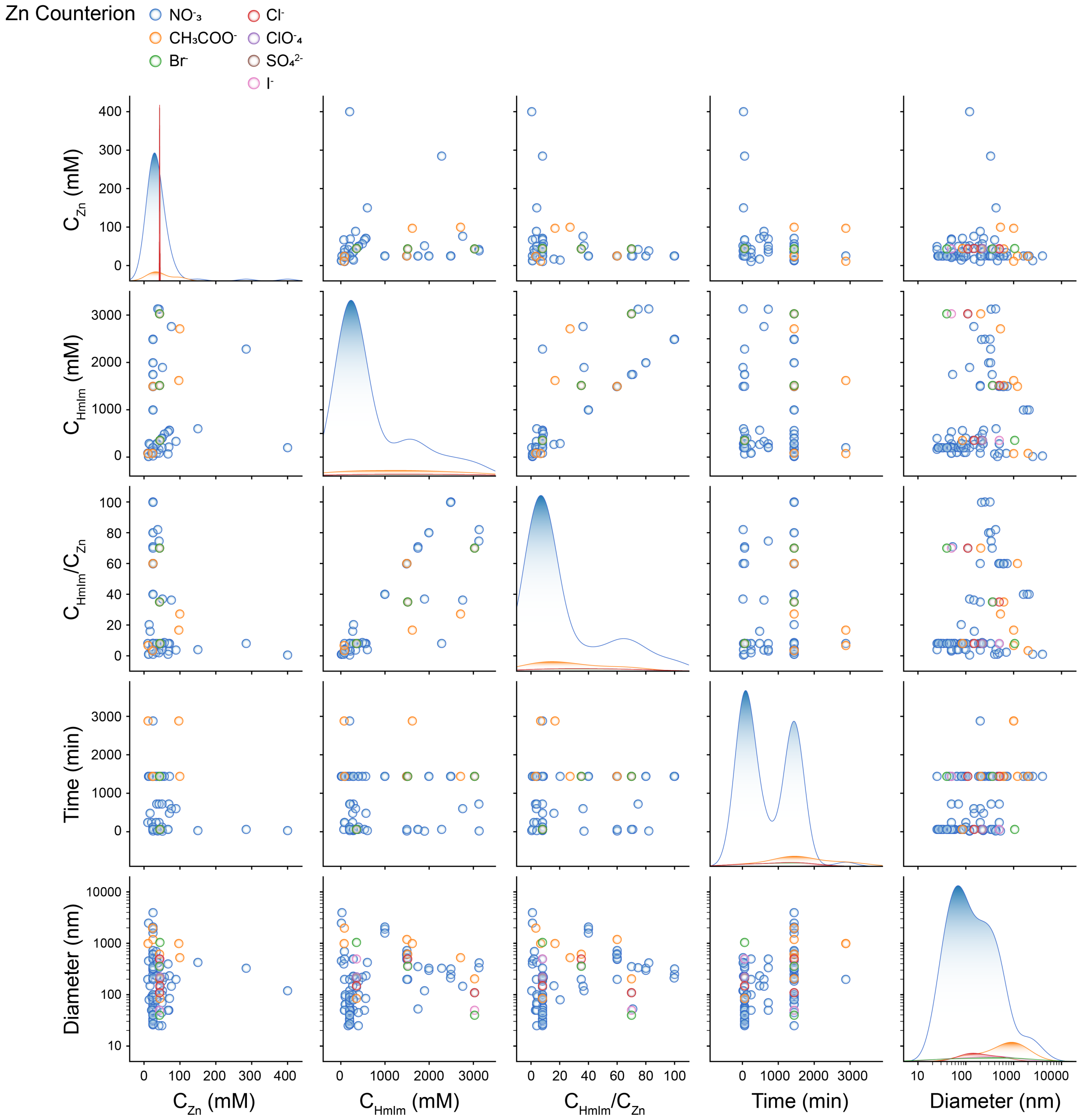

Figure S8: Pair plot for different synthetic variables, classify according to the counterion employed.

BET area trends and Diameter-shape correlation

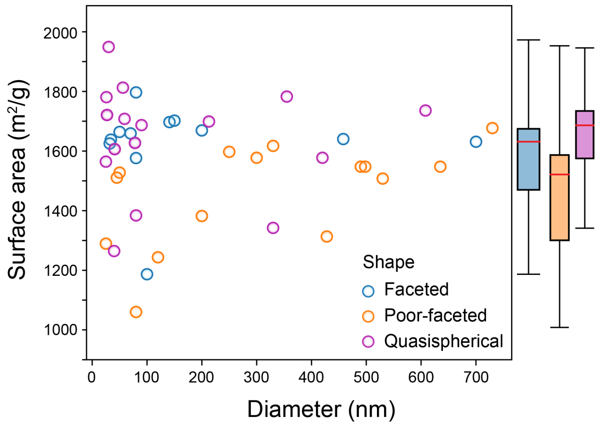

Figure S9: BET surface area cross dependence with particle diameter, classify by morphology. Box plots on the right represent the distribution of surface area values by morphology, considering all sizes.

From the 254 entries after filtering the data set as described above, only 101 reported a value for the surface area. From this sub-set, reported values below 1000 m^2g^{-1} (which represents about 50% of the estimated crystallographic porosity value of 1947 m^2g^{-1})[S45] were discarded. The remaining 83 entries were classify according to the morphology of the particles. Figure S9 cross-references the reported BET surface areas with particles' diameter.

Partial dependence plots

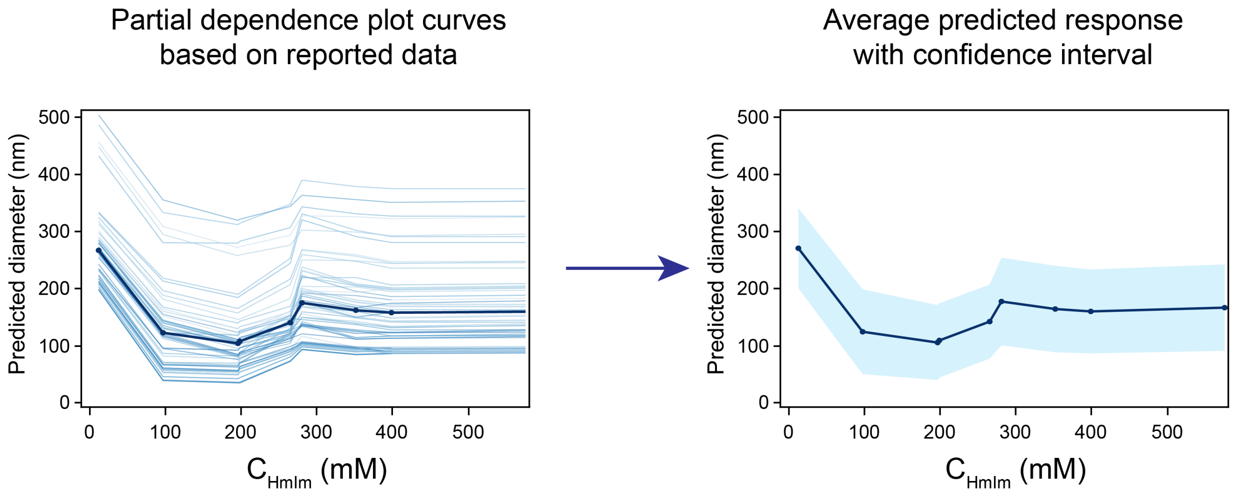

Figure S10: Exemplification of obtaining an average predicted response with a confidence interval based on predicted curves in partial dependence plots.

A partial dependence plot (PDP) is a graphical representation of the relationship between a feature and the predicted outcome of a machine learning model while holding all other features constant. For each data point, the value of one feature is varied while the values of all other features are held constant. The predicted outcome is plotted against the varied feature values. It is a useful tool for understanding how individual features contribute to the overall prediction of a model. The shape of the plot can be used to understand how the feature affects the prediction. For example, a linear relationship would indicate that the feature has a proportional effect on the prediction. A non-linear relationship would indicate that the feature has a more complex effect on the prediction.

References

(S1) Allegretto, J. A.; Onna, D.; Acion, L.; Bilmes, S. A.; Azzaroni, O.; Rafti, M. Dataset of reported synthetic conditions for ZIF-8 since 2006 and final characteristics and properties of the particles obtained. 2023; `https://doi.org/10.5281/zenodo.8153451`.

(S2) Cravillon, J.; Nayuk, R.; Springer, S.; Feldhoff, A.; Huber, K.; Wiebcke, M. Controlling Zeolitic Imidazolate Framework Nano- and Microcrystal Formation: Insight into Crystal Growth by Time-Resolved In Situ Static Light Scattering. *Chemistry of Materials* **2011**, *23*, 2130–2141.

(S3) Kida, K.; Okita, M.; Fujita, K.; Tanaka, S.; Miyake, Y. Formation of high crystalline ZIF-8 in an aqueous solution. *CrystEngComm* **2013**, *15*, 1794.

(S4) Weber, M. D.; Baker, T. L.; Dao, B.; Kwon, C.; Tian, F. Exploring the Aggregative Growth of Nanoporous Zeolitic Imidazolate Framework ZIF-8. *Crystal Growth and Design* **2020**, *20*, 2305–2312.

(S5) Böhme, U.; Barth, B.; Paula, C.; Kuhnt, A.; Schwieger, W.; Mundstock, A.; Caro, J.; Hartmann, M. Ethene/Ethane and Propene/Propane Separation via the Olefin and Paraffin Selective Metal–Organic Framework Adsorbents CPO-27 and ZIF-8. *Langmuir* **2013**, *29*, 8592–8600.

(S6) Bustamante, E. L.; Fernández, J. L.; Zamaro, J. M. Influence of the solvent in the synthesis of zeolitic imidazolate framework-8 (ZIF-8) nanocrystals at room temperature. *Journal of Colloid and Interface Science* **2014**, *424*, 37–43.

(S7) Chang, N.; Gu, Z.-Y.; Yan, X.-P. Zeolitic Imidazolate Framework-8 Nanocrystal Coated Capillary for Molecular Sieving of Branched Alkanes from Linear Alkanes along with High-Resolution Chromatographic Separation of Linear Alkanes. *Journal of the American Chemical Society* **2010**, *132*, 13645–13647.

(S8) Cheng, L.; Yan, P.; Yang, X.; Zou, H.; Yang, H.; Liang, H. Microstructure controlling and dielectric relaxation dynamic of ZIF-8 synthesized in different solvents. *Materials Chemistry and Physics* **2020**, *247*, 122869.

(S9) Cravillon, J.; Münzer, S.; Lohmeier, S.-J.; Feldhoff, A.; Huber, K.; Wiebcke, M. Rapid

Room-Temperature Synthesis and Characterization of Nanocrystals of a Prototypical Zeolitic Imidazolate Framework. *Chemistry of Materials* **2009**, *21*, 1410–1412.

(S10) Cravillon, J.; Schröder, C. A.; Nayuk, R.; Gummel, J.; Huber, K.; Wiebcke, M. Fast Nucleation and Growth of ZIF-8 Nanocrystals Monitored by Time-Resolved In Situ Small-Angle and Wide-Angle X-Ray Scattering. *Angewandte Chemie International Edition* **2011**, *50*, 8067–8071.

(S11) He, M.; Yao, J.; Liu, Q.; Wang, K.; Chen, F.; Wang, H. Facile synthesis of zeolitic imidazolate framework-8 from a concentrated aqueous solution. *Microporous and Mesoporous Materials* **2014**, *184*, 55–60.

(S12) He, S.; Wang, H.; Zhang, C.; Zhang, S.; Yu, Y.; Lee, Y.; Li, T. A generalizable method for the construction of MOF@polymer functional composites through surface-initiated atom transfer radical polymerization. *Chemical Science* **2019**, *10*, 1816–1822.

(S13) Jayaramulu, K.; Datta, K. K. R.; Rösler, C.; Petr, M.; Otyepka, M.; Zboril, R.; Fischer, R. A. Biomimetic Superhydrophobic/Superoleophilic Highly Fluorinated Graphene Oxide and ZIF-8 Composites for Oil-Water Separation. *Angewandte Chemie International Edition* **2016**, *55*, 1178–1182.

(S14) Jian, M.; Liu, B.; Liu, R.; Qu, J.; Wang, H.; Zhang, X. Water-based synthesis of zeolitic imidazolate framework-8 with high morphology level at room temperature. *RSC Advances* **2015**, *5*, 48433–48441.

(S15) Jiang, Y.; Liu, H.; Tan, X.; Guo, L.; Zhang, J.; Liu, S.; Guo, Y.; Zhang, J.; Wang, H.; Chu, W. Monoclinic ZIF-8 Nanosheet-Derived 2D Carbon Nanosheets as Sulfur Immobilizer for High-Performance Lithium Sulfur Batteries. *ACS Applied Materials and Interfaces* **2017**, *9*, 25239–25249.

(S16) Khan, N. A.; Jung, B. K.; Hasan, Z.; Jhung, S. H. Adsorption and removal of phthalic

acid and diethyl phthalate from water with zeolitic imidazolate and metal-organic frameworks. *Journal of Hazardous Materials* **2015**, *282*, 194–200.

(S17) Lai, L. S.; Yeong, Y. F.; Ani, N. C.; Lau, K. K.; Shariff, A. M. Effect of synthesis parameters on the formation of zeolitic imidazolate framework 8 (ZIF-8) nanoparticles for CO2 adsorption. *Particulate Science and Technology* **2014**, *32*, 520–528.

(S18) Lai, Q.; Zheng, L.; Liang, Y.; He, J.; Zhao, J.; Chen, J. Metal-Organic-Framework-Derived Fe-N/C Electrocatalyst with Five-Coordinated Fe-Nx Sites for Advanced Oxygen Reduction in Acid Media. *ACS Catalysis* **2017**, *7*, 1655–1663.

(S19) Lee, Y. R.; Jang, M. S.; Cho, H. Y.; Kwon, H. J.; Kim, S.; Ahn, W. S. ZIF-8: A comparison of synthesis methods. *Chemical Engineering Journal* **2015**, *271*, 276–280.

(S20) Lee, T.; Kim, H.; Cho, W.; Han, D. Y.; Ridwan, M.; Yoon, C. W.; Lee, J. S.; Choi, N.; Ha, K. S.; Yip, A. C.; Choi, J. Thermosensitive structural changes and adsorption properties of zeolitic imidazolate framework-8 (ZIF-8). *Journal of Physical Chemistry C* **2015**, *119*, 8226–8237.

(S21) Li, J.; Wu, Y. N.; Li, Z.; Zhang, B.; Zhu, M.; Hu, X.; Zhang, Y.; Li, F. Zeolitic imidazolate framework-8 with high efficiency in trace arsenate adsorption and removal from water. *Journal of Physical Chemistry C* **2014**, *118*, 27382–27387.

(S22) Li, X.; Hao, C.; Tang, B.; Wang, Y.; Liu, M.; Wang, Y.; Zhu, Y.; Lu, C.; Tang, Z. Supercapacitor electrode materials with hierarchically structured pores from carbonization of MWCNTs and ZIF-8 composites. *Nanoscale* **2017**, *9*, 2178–2187.

(S23) Lu, G.; Li, S.; Guo, Z.; Farha, O. K.; Hauser, B. G.; Qi, X.; Wang, Y.; Wang, X.; Han, S.; Liu, X.; Duchene, J. S.; Zhang, H.; Zhang, Q.; Chen, X.; Ma, J.; Loo, S. C. J.; Wei, W. D.; Yang, Y.; Hupp, J. T.; Huo, F. Imparting functionality to a metal-organic framework material by controlled nanoparticle encapsulation. *Nature Chemistry* **2012**, *4*, 310–316.

(S24) Pan, Y.; Liu, Y.; Zeng, G.; Zhao, L.; Lai, Z. Rapid synthesis of zeolitic imidazolate framework-8 (ZIF-8) nanocrystals in an aqueous system. *Chemical Communications* **2011**, *47*, 2071.

(S25) Saghir, S.; Xiao, Z. Facile preparation of metal-organic frameworks-8 (ZIF-8) and its simultaneous adsorption of tetracycline (TC) and minocycline (MC) from aqueous solutions. *Materials Research Bulletin* **2021**, *141*, 111372.

(S26) Schejn, A.; Balan, L.; Falk, V.; Aranda, L.; Medjahdi, G.; Schneider, R. Controlling ZIF-8 nano- and microcrystal formation and reactivity through zinc salt variations. *CrystEngComm* **2014**, *16*, 4493–4500.

(S27) Shen, K.; Zhang, L.; Chen, X.; Liu, L.; Zhang, D.; Han, Y.; Chen, J.; Long, J.; Luque, R.; Li, Y.; Chen, B. Ordered macro-microporous metal-organic framework single crystals. *Science* **2018**, *359*, 206–210.

(S28) Tran, U. P.; Le, K. K.; Phan, N. T. Expanding applications of metal-organic frameworks: Zeolite imidazolate framework zif-8 as an efficient heterogeneous catalyst for the knoevenagel reaction. *ACS Catalysis* **2011**, *1*, 120–127.

(S29) Tsai, C.-W.; Langner, E. H. The effect of synthesis temperature on the particle size of nano-ZIF-8. *Microporous and Mesoporous Materials* **2016**, *221*, 8–13.

(S30) Venna, S. R.; Jasinski, J. B.; Carreon, M. A. Structural Evolution of Zeolitic Imidazolate Framework-8. *Journal of the American Chemical Society* **2010**, *132*, 18030–18033.

(S31) Wang, Z.; Jin, H.; Meng, T.; Liao, K.; Meng, W.; Yang, J.; He, D.; Xiong, Y.; Mu, S. Fe, Cu-Coordinated ZIF-Derived Carbon Framework for Efficient Oxygen Reduction Reaction and Zinc-Air Batteries. *Advanced Functional Materials* **2018**, *28*, 1802596.

(S32) Wu, Y. N.; Zhou, M.; Zhang, B.; Wu, B.; Li, J.; Qiao, J.; Guan, X.; Li, F. Amino

acid assisted templating synthesis of hierarchical zeolitic imidazolate framework-8 for efficient arsenate removal. *Nanoscale* **2014**, *6*, 1105–1112.

(S33) Zhang, C.; Lively, R. P.; Zhang, K.; Johnson, J. R.; Karvan, O.; Koros, W. J. Unexpected Molecular Sieving Properties of Zeolitic Imidazolate Framework-8. *The Journal of Physical Chemistry Letters* **2012**, *3*, 2130–2134.

(S34) Zhang, K.; Lively, R. P.; Zhang, C.; Chance, R. R.; Koros, W. J.; Sholl, D. S.; Nair, S. Exploring the framework hydrophobicity and flexibility of zif-8: From biofuel recovery to hydrocarbon separations. *Journal of Physical Chemistry Letters* **2013**, *4*, 3618–3622.

(S35) Zhang, W.; Wu, Z.-Y.; Jiang, H.-L.; Yu, S.-H. Nanowire-Directed Templating Synthesis of Metal–Organic Framework Nanofibers and Their Derived Porous Doped Carbon Nanofibers for Enhanced Electrocatalysis. *Journal of the American Chemical Society* **2014**, *136*, 14385–14388.

(S36) Zheng, H.; Zhang, Y.; Liu, L.; Wan, W.; Guo, P.; Nyström, A. M.; Zou, X. One-pot Synthesis of Metal–Organic Frameworks with Encapsulated Target Molecules and Their Applications for Controlled Drug Delivery. *Journal of the American Chemical Society* **2016**, *138*, 962–968.

(S37) Zhou, K.; Mousavi, B.; Luo, Z.; Phatanasri, S.; Chaemchuen, S.; Verpoort, F. Characterization and properties of Zn/Co zeolitic imidazolate frameworks vs. ZIF-8 and ZIF-67. *Journal of Materials Chemistry A* **2017**, *5*, 952–957.

(S38) Zhu, M.; Srinivas, D.; Bhogeswararao, S.; Ratnasamy, P.; Carreon, M. A. Catalytic activity of ZIF-8 in the synthesis of styrene carbonate from CO2 and styrene oxide. *Catalysis Communications* **2013**, *32*, 36–40.

(S39) Zhu, Y.; Ciston, J.; Zheng, B.; Miao, X.; Czarnik, C.; Pan, Y.; Sougrat, R.; Lai, Z.; Hsiung, C. E.; Yao, K.; Pinnau, I.; Pan, M.; Han, Y. Unravelling surface and interfacial

structures of a metal-organic framework by transmission electron microscopy. *Nature Materials* **2017**, *16*, 532–536.

(S40) Mutaftschiev, B.; Mutaftschiev, B. *The atomistic nature of crystal growth*; Springer, 2001; Vol. 43.

(S41) Pierre, A. C. *Introduction to sol-gel processing*; Springer Nature, 2020.

(S42) Karthika, S.; Radhakrishnan, T. K.; Kalaichelvi, P. A Review of Classical and Non-classical Nucleation Theories. *Crystal Growth and Design* **2016**, *16*, 6663–6681.

(S43) Van Vleet, M. J.; Weng, T.; Li, X.; Schmidt, J. R. In Situ, Time-Resolved, and Mechanistic Studies of Metal-Organic Framework Nucleation and Growth. *Chemical Reviews* **2018**, *118*, 3681–3721.

(S44) Aerts, A.; Kirschhock, C. E.; Martens, J. A. Methods for in situ spectroscopic probing of the synthesis of a zeolite. *Chemical Society Reviews* **2010**, *39*, 4626–4642.

(S45) Park, K. S.; Ni, Z.; Cote, A. P.; Choi, J. Y.; Huang, R.; Uribe-Romo, F. J.; Chae, H. K.; O'Keeffe, M.; Yaghi, O. M. Exceptional chemical and thermal stability of zeolitic imidazolate frameworks. *Proceedings of the National Academy of Sciences* **2006**, *103*, 10186–10191.
